# Strategies to Overcome Hurdles in Cancer Immunotherapy

**DOI:** 10.34133/bmr.0080

**Published:** 2024-09-19

**Authors:** Jihyun Kim, Byung Joon Lee, Sehoon Moon, Hojeong Lee, Juyong Lee, Byung-Soo Kim, Keehoon Jung, Hyungseok Seo, Yeonseok Chung

**Affiliations:** ^1^ Research Institute for Pharmaceutical Sciences, College of Pharmacy, College of Pharmacy,Seoul National University, Seoul 08826, Republic of Korea.; ^2^ Interdisciplinary Program for Bioengineering, Seoul National University, Seoul 08826, Republic of Korea.; ^3^ Department of Anatomy and Cell Biology, Department of Biomedical Sciences, Seoul National University College of Medicine, Seoul 03080, Republic of Korea.; ^4^ Molecular Medicine and Biopharmaceutical Sciences, Graduate School of Convergence Science and Technology, Seoul National University, Seoul 08826, Republic of Korea.; ^5^ Arontier Co., Seoul 06735, Republic of Korea.; ^6^ School of Chemical and Biological Engineering, Seoul National University, Seoul 08826, Republic of Korea.; ^7^ Institute of Chemical Processes, Institute of Engineering Research, and BioMAX, Seoul National University, Seoul 08826, Republic of Korea.

## Abstract

Despite marked advancements in cancer immunotherapy over the past few decades, there remains an urgent need to develop more effective treatments in humans. This review explores strategies to overcome hurdles in cancer immunotherapy, leveraging innovative technologies including multi-specific antibodies, chimeric antigen receptor (CAR) T cells, myeloid cells, cancer-associated fibroblasts, artificial intelligence (AI)-predicted neoantigens, autologous vaccines, and mRNA vaccines. These approaches aim to address the diverse facets and interactions of tumors’ immune evasion mechanisms. Specifically, multi-specific antibodies and CAR T cells enhance interactions with tumor cells, bolstering immune responses to facilitate tumor infiltration and destruction. Modulation of myeloid cells and cancer-associated fibroblasts targets the tumor’s immunosuppressive microenvironment, enhancing immunotherapy efficacy. AI-predicted neoantigens swiftly and accurately identify antigen targets, which can facilitate the development of personalized anticancer vaccines. Additionally, autologous and mRNA vaccines activate individuals’ immune systems, fostering sustained immune responses against cancer neoantigens as therapeutic vaccines. Collectively, these strategies are expected to enhance efficacy of cancer immunotherapy, opening new horizons in anticancer treatment.

## Introduction

Recent advancements in cancer treatment have profoundly altered the therapeutic landscape, offering new hope and improved outcomes for patients worldwide. Breakthroughs in precision medicine, immunotherapy, and targeted therapies have revolutionized how we approach cancer care. Precision medicine, driven by advancements in genomic sequencing and molecular profiling, allows for tailored treatment strategies based on an individual’s genetic makeup and tumor characteristics. Immunotherapy, including immune checkpoint inhibitors and chimeric antigen receptor (CAR) T cell therapy, harnesses the power of the immune system to recognize and eradicate cancer cells. Additionally, targeted therapies specifically target molecules involved in tumor growth and progression, minimizing damage to healthy cells.

Despite remarkable advancements in recent decades, significant challenges persist in achieving widespread efficacy across diverse cancer types and patient populations. This underscores the urgent need for further research and development to enhance the effectiveness of cancer immunotherapies. In this review, we explore the strategies aimed at overcoming hurdles in cancer immunotherapy, focusing on 5 innovative approaches that harness the power of the immune system to target and eliminate tumors. These technologies may offer unprecedented opportunities to refine treatment strategies and address the complexities of tumor–immune interactions. By elucidating these innovative approaches, this review aims to provide insights into the evolving landscape of cancer immunotherapy and highlight avenues for future research and clinical translation.

## Multi-Specific Antibodies and CAR Therapy

In the rapidly evolving field of cancer immunotherapy, 2 innovative approaches have notably emerged: multi-specific antibodies and CAR T cell therapy [1–3]. Multi-specific antibodies, with their ability to recognize 2 or more epitopes on the same or distinct targets, represent a significant leap beyond traditional monoclonal antibodies or cytokine therapies [1,2,4]. The field has seen a progressive evolution from the initial focus on bispecific antibodies to the Food and Drug Administration (FDA) approval of BiTE (bispecific T cell engager) therapies and, most recently, the exploration into trispecific antibodies and conjugated antibodies that combine drugs or cytokines for enhanced therapeutic effects [5].

On the parallel front of immunotherapy, CAR T cell therapy has made groundbreaking strides in treating hematologic malignancies [6–8]. Engineered to redirect T cells to tumor-specific antigens (TSAs), CAR T cell treatments have shown remarkable success, particularly against relapsed and/or refractory B cell lymphomas, acute lymphoblastic leukemia, and multiple myeloma [9,10]. Currently, 6 FDA-approved CAR T cell therapies targeting CD19 and B cell maturation antigen (BCMA) are utilized for treating hematologic cancers, and there is ongoing development of CAR T cell therapies aimed at solid tumors and other severe diseases [11].

In this article, we highlight the advancements and challenges in multi-specific antibody and CAR T cell therapy, with a focus on exploring their mechanisms and strategies for overcoming obstacles, emphasizing their future role in cancer treatment

### Mechanisms and challenges of multi-specific antibodies

Over the past 10 years, immune checkpoint blockade (ICB) therapy has emerged as one of the most clinically effective immunotherapies. Blocking of immune checkpoints such as PD-1 or CTLA-4 on T cells prevents T cell exhaustion and prolongs the activation state. Currently, FDA-approved checkpoint blockade antibodies cover 20 cancer types, including melanoma, non-small cell lung cancer (NSCLC), renal cell carcinoma (RCC), and urothelial carcinoma (UC) [12]. However, the major challenge of these antibodies is the low response rate [13]. On average, only 20 to 30% of patients achieve clinical benefit on average when treated with a single checkpoint blockade antibody [13–16]. Many studies have shown that combination with other antibodies with different checkpoint targets or chemotherapies enhances the efficacy of ICB immunotherapy [14,17–19].

**Fig. 1.**
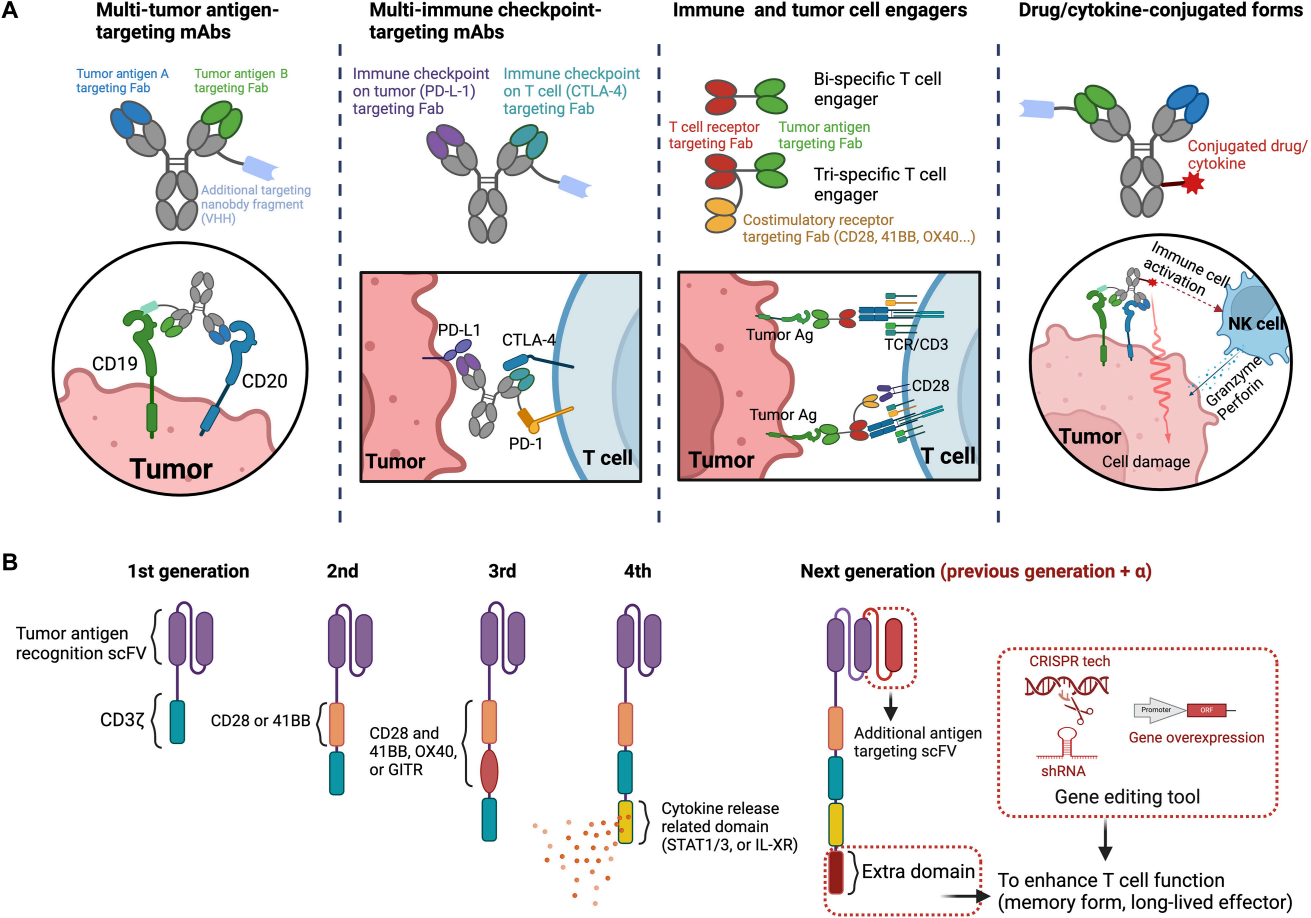
Scheme of next-generation multi-specific antibodies and CAR T therapies. (A) Illustration of the diversity and function of multi-specific antibodies. Multi-tumor antigen-targeting mAbs: These are illustrated with domains that bind to various TSAs, like CD19 and CD20, potentially integrating an extra nanobody fragment for enhanced targeting. Multi-immune checkpoint-targeting mAbs: Highlighted are antibodies designed to engage with several immune checkpoints simultaneously, such as PD-L1 on tumors and CTLA-4 on T cells, modulating the immune system’s response to cancer. Immune and tumor cell engagers: These engagers are depicted as bridging immune cells with cancer cells, promoting interactions that can amplify the body’s immune-mediated attack on tumors. Drug/cytokine-conjugated forms: This section shows antibodies conjugated with drugs or cytokines, linking therapeutic agents directly to the antibody structure to bolster antitumor effects. (B) Five generations of CAR T cell design. The first generation contains a simple structure with only the CD3ζ signaling domain. The second and third generations add one or more costimulatory domains (e.g., CD28 and 4-1BB) to the CAR structure for improved cell activation and persistence. The fourth generation incorporates a cytokine release domain to further enhance the immune response. The next generation of CAR T includes additional modifications such as extra antigen-targeting domains and gene editing tools like CRISPR to augment T cell function and persistence. This panel exemplifies the advancements in CAR T therapy aimed at enhancing specificity, efficacy, and long-term immune memory in cancer treatment.

Multi-specific antibodies have the potential to overcome the current limitations of ICB immunotherapy. Multi-specific antibodies, with their capacity to target multiple antigens simultaneously, mark a notable evolution from traditional monoclonal antibodies that are limited to single-antigen binding [4,20]. Here, we elucidate several key biological mechanisms that underpin the functioning of multi-specific antibodies (Fig. 1A). Multi-specific antibodies currently approved by the FDA or in clinical trials have been classified according to their mechanism of action [21–24] (Table S1).

#### Multi-tumor antigen-targeting monoclonal antibodies

Multi-specific antibodies are engineered through recombinant DNA technology by fusing genes for different antibody binding domains [1,4,20]. This results in molecules with multiple antigen-binding sites for targeting various antigens. At the molecular level, multi-specific antibodies can engage with multiple antigens on the cancer cells. For example, amivantamab binds to both epidermal growth factor receptor (EGFR) and c-Met on NSCLC cell membrane and blocks the cell proliferation signal, which is provided by their ligands EGF and hepatocyte growth factor (HGF) [25].

It is also possible to bind different epitopes of one antigen. Zanidatamab, a biparatopic antibody, has nonoverlapping epitopes of the HER-2 antigen. This engagement allows antibodies to bind to cancer cells with low antigen expression [26].

#### Multi-immune checkpoint-targeting monoclonal antibodies

Beyond direct cell targeting, multi-specific antibodies can modulate signal transduction pathways within cells. By binding to specific receptors or ligands, these antibodies can either block inhibitory signals that cancer cells use to evade the immune response or stimulate activating signals that enhance the immune response against cancer cells [27]. Cadonilimab, a PD-1/CTLA-4 bispecific antibody, blocks exhaustion signaling on T cells to enhance T cell function.

#### Immune cell engager

Multi-specific antibodies can simultaneously engage with multiple antigens on the surface of cancer cells and immune cells. For instance, a bispecific antibody might have one arm that binds to CD19 on B cells and another arm that binds to CD3 on T cells. This dual engagement brings T cells into proximity with cancer cells, promoting T cell activation and the targeted killing of the cancer cells [28,29].

Some multi-specific antibodies are designed to recruit other components of the immune system, such as macrophages or natural killer (NK) cells, to the tumor site. This is achieved by including binding domains that engage with Fc receptors on immune cells, triggering phagocytosis or the release of cytotoxic granules that lead to tumor cell death [30]. The versatility of multi-specific antibodies allows for the targeting of heterogenous tumor cell populations. By engaging multiple antigens, these antibodies can overcome the challenges posed by tumor antigen variability and escape mechanisms.

#### Drug/cytokine or its receptor-conjugated antibodies

The engineering flexibility of multi-specific antibodies enables the incorporation of various functional modules, including cytokine payloads or enzyme inhibitors, to enhance therapeutic efficacy [5,21,22]. Bintrafusp alfa, a bispecific antibody targeting PD-L1/transforming growth factor β (TGFβ), binds to PD-L1 on cancer cells to block PD-1/PD-L1 interaction with T cells leading to T cell exhaustion, and captures TGFβ, blocking downstream immunosuppressive signals with the other arm [22]. This antibody enhances its efficacy by avoiding an immunosuppressive environment with cytokine receptors and immune checkpoint inhibitors.

#### Remaining challenges

The development of multi-specific antibodies, despite their therapeutic promise, encounters significant challenges. Engineering these antibodies is more complex than traditional monoclonal antibodies, requiring innovative techniques for correct folding and assembly. They may face stability issues and poor pharmacokinetics, leading to rapid clearance or reduced efficacy [31]. Immunogenicity poses additional risks, potentially triggering immune responses that diminish therapy effectiveness. Target selection is crucial, and penetrating solid tumors is particularly challenging, complicating their use against solid cancers. The multiplicity of targets also complicates therapeutic window prediction and increases the risk of off-target effects and toxicity, demanding careful design and extensive preclinical testing to minimize adverse outcomes [32].

Despite these challenges, the therapeutic potential of multi-specific antibodies continues to drive innovation in their development. The ongoing research and clinical trials in this area are closely watched by the scientific and medical communities, with the hope that multi-specific antibodies will play a crucial role in the future of oncology (Fig. 1A).

### Mechanisms and challenges of CAR T cell therapy

The mechanism of CAR T cell therapy is predicated on the modification of T cells to express CARs, which are synthetic receptors that confer specificity against cancer cell antigens (Fig. 1B). A CAR is composed of an extracellular antigen-binding domain, a hinge region, a transmembrane domain, and an intracellular signaling domain. The antigen-binding domain is typically derived from the variable regions of an antibody specific to a tumor antigen, enabling the recognition of cancer cells. The intracellular domain usually contains the CD3ζ chain responsible for initiating T cell activation, often combined with one or more costimulatory domains (e.g., CD28 and 4-1BB) to enhance T cell proliferation, persistence, and cytotoxic function [9,29]. Currently, CAR T cells are generated by transferring genes into T cells using lentivirus or retrovirus vectors, allowing these cells to express CARs that target and destroy cancer cells. After infusion into the patient, CAR T cells eliminate cancer cells, and a portion of these engineered cells can transform into memory T cells, offering prolonged immunity. To enhance the longevity and efficacy of the memory T cells, some approaches involve knocking out/in specific genes in the CAR T cells, thereby strengthening their persistence and functional capabilities [33–35]. This genetic modification aims to improve the overall success and durability of CAR T cell therapy in treating cancer.

While CAR T cell therapy has shown remarkable success, particularly in treating certain blood cancers, several challenges limit its broader application and effectiveness. One of the most significant side effects of CAR T cell therapy is cytokine release syndrome (CRS), a systemic inflammatory response caused by the rapid proliferation of CAR T cells and the subsequent release of cytokines. CRS can range from mild flu-like symptoms to severe, life-threatening conditions [32]. Another serious side effect is neurotoxicity, which can manifest as confusion, seizures, or severe encephalopathy. The exact mechanism is not fully understood, making it a critical area of ongoing research [32]. CAR T cell therapy has been most effective against hematologic malignancies. Its efficacy against solid tumors is limited by several factors, including the tumor microenvironment’s (TME) suppressive nature, the heterogeneity of antigen expression on solid tumors, and the physical barriers that impede CAR T cell infiltration into the tumor. Moreover, tumor cells can evade CAR T cell therapy by down-regulating or losing the target antigen. This antigen escape mechanism can lead to relapse and the need for alternative strategies to target the cancer cells [6]. The production of CAR T cells is a complex, time-consuming, and costly process that requires specialized facilities. The personalized nature of the therapy also presents logistical challenges, including the need for rapid transportation of cells between the patient and the manufacturing site.

Despite these challenges, CAR T cell therapy represents a significant advancement in cancer treatment. Ongoing research is focused on addressing these challenges through the development of new CAR designs, strategies to manage side effects, and approaches to enhance the efficacy of CAR T cells against solid tumors (Table S2). The continuous evolution of CAR T cell technology holds promise for expanding its applicability and improving outcomes for patients with various types of cancer (Fig. 1B).

Recently, multi-specific antibodies have been combined with CAR therapy to improve the ability to target tumor-associated antigens (TAAs). In addition to improving efficacy by altering the intracellular domain of the CAR, the extracellular antigen-targeting region is also being engineered for better contact. Additional scFv or VHH regions next to conventional scFv with different structures could be an example. Logic gate strategies are also used in cancer immunotherapy to minimize the impact on the normal tissues and to overcome tumor heterogeneity.

## Targeting CAFs and Myeloid Cells in TME

The TME orchestrates a complex interplay between cancer cells and various stromal components, among which cancer-associated fibroblasts (CAFs) and myeloid cells emerge as key players [36,37]. CAFs, a heterogeneous population of cells derived from resident fibroblasts or recruited from other sources like bone marrow-derived mesenchymal stem cells, encompass distinct subsets including myofibroblastic CAFs (myCAFs), inflammatory CAFs (iCAFs), and antigen-presenting CAFs (apCAFs) [38]. Each subset exhibits unique characteristics and functions within the TME.

### The roles of CAFs and myeloid cells in TME

**Fig. 2.**
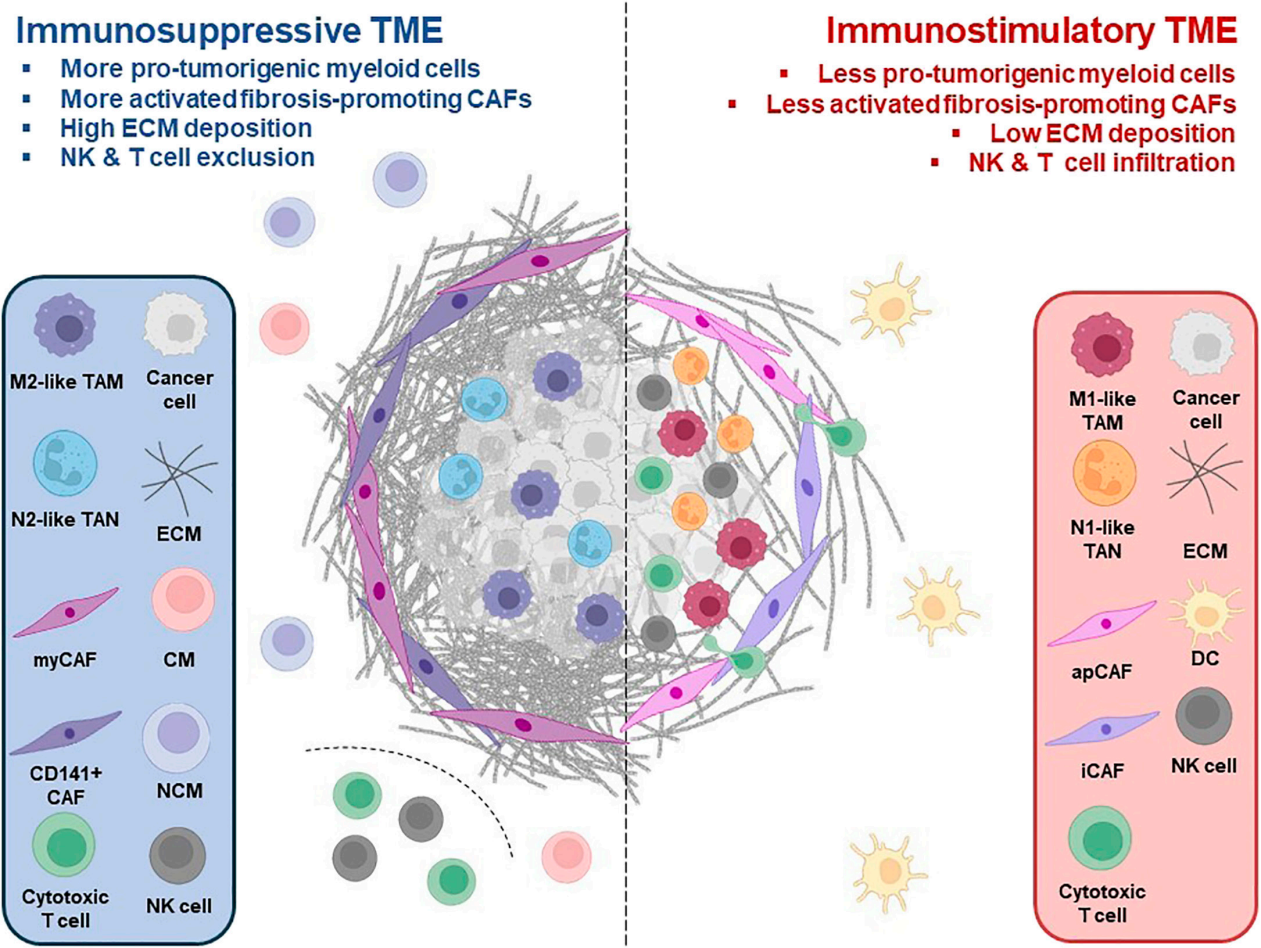
Versatile CAFs and myeloid cells inhabit various TMEs. The immunosuppressive TME is characterized by the typical infiltration of pro-tumorigenic monocyte subsets, M2-like TAMs, and N2-like TANs, along with activated fibrosis-promoting CAFs that induce ECM deposition. This ECM deposition can serve as a physical barrier to tumor-infiltrating T and NK cells. On the other hand, an immunostimulatory TME generally shows less ECM deposition and a low number of pro-tumorigenic myeloid cells, resulting in abundant infiltration of cytotoxic T cells. TAN, tumor-associated neutrophil; TAM, tumor-associated macrophage; CM, classical monocyte; NCM, nonclassical monocyte; apCAF, antigen-presenting CAF; myCAF, myofibroblastic CAF; iCAF, inflammatory CAF; ECM, extracellular matrix. Depending on the context, classical monocytes, nonclassical monocytes, iCAFs, and apCAFs can also act as immunostimulatory cell subtypes.

The myCAFs contribute to extracellular matrix (ECM) remodeling and fibrosis within the TME. They play crucial roles in promoting tumor invasion and metastasis through the deposition of collagen and other ECM components [39]. The iCAFs are distinguished by their expression of inflammatory mediators and cytokines. iCAFs contribute to the establishment of an immunosuppressive TME by promoting the recruitment and activation of immunosuppressive immune cells, such as regulatory T cells and myeloid-derived suppressor cells (MDSCs). The apCAFs represent a unique subset of CAFs capable of presenting antigens to immune cells within the TME [40]. By expressing major histocompatibility complex (MHC) class II molecules and costimulatory molecules, apCAFs facilitate the activation of tumor-infiltrating lymphocytes (TILs) and promote antitumor immune responses. Together, myCAFs, iCAFs, and apCAFs constitute key components of the tumor stroma, exerting multifaceted effects on tumor progression, therapy response, and immune evasion [36]. Recently, CD141^+^ CAFs were also identified, and they are known to be involved in tumor fibrosis in pancreatic cancer [40]. Thus, understanding the distinct functions and interactions of these CAF subsets within the TME is crucial for the development of targeted therapeutic strategies aimed at disrupting their pro-tumorigenic activities (Fig. 2).

Conversely, myeloid cells, including monocytes (comprising classical, intermediate, and nonclassical subsets), neutrophils, and predominantly tumor-associated macrophages (TAMs) and so-called MDSCs, profoundly influence the TME’s immunosuppressive milieu [41]. Monocytes, recruited to the tumor site from the bloodstream, can differentiate into TAMs or directly contribute to immune suppression [37,42,43]. TAMs, often polarized to an M2-like phenotype, dampen antitumor immunity through various mechanisms. Neutrophils, another critical component of the myeloid lineage, play multifaceted roles in tumor progression. While traditionally viewed as effector cells in acute inflammation (i.e., N1-like phenotype), neutrophils can also promote tumor growth and metastasis through the release of pro-angiogenic factors and suppression of immune responses (i.e., N2-like phenotype) [44] (Fig. 2).

### Strategies to target CAFs and myeloid cells within TME

Based on the diverse roles that CAFs and myeloid cells play within the TME, several strategies have been proposed to selectively target CAFs and mitigate their pro-tumorigenic effects. One approach involves direct inhibition of signaling pathways implicated in CAF activation and function. For instance, targeting the TGFβ signaling pathway, a key driver of CAF activation and ECM remodeling, holds promise in restraining CAF-mediated tumor progression [45]. Additionally, strategies aimed at depleting CAF populations have been explored, including the use of targeted antibodies against CAF-specific markers such as fibroblast activation protein. Moreover, emerging therapeutic modalities seek to reprogram CAFs toward a quiescent, tumor-restraining phenotype by modulating signaling pathways involved in CAF activation or by targeting metabolic vulnerabilities unique to activated CAFs [36].

Targeting myeloid cells within the TME represents a promising avenue for enhancing antitumor immunity and overcoming immunosuppressive barriers [37]. One strategy involves disrupting the recruitment of myeloid cells into tumors by targeting chemokine receptors or their ligands critical for myeloid cell trafficking [43]. Moreover, targeting signaling pathways implicated in myeloid cell activation, such as CSF-1/CSF-1R axis inhibition, represents a promising strategy to mitigate their immunosuppressive effects and restore antitumor immunity [46] (Fig. 2).

**Fig. 3.**
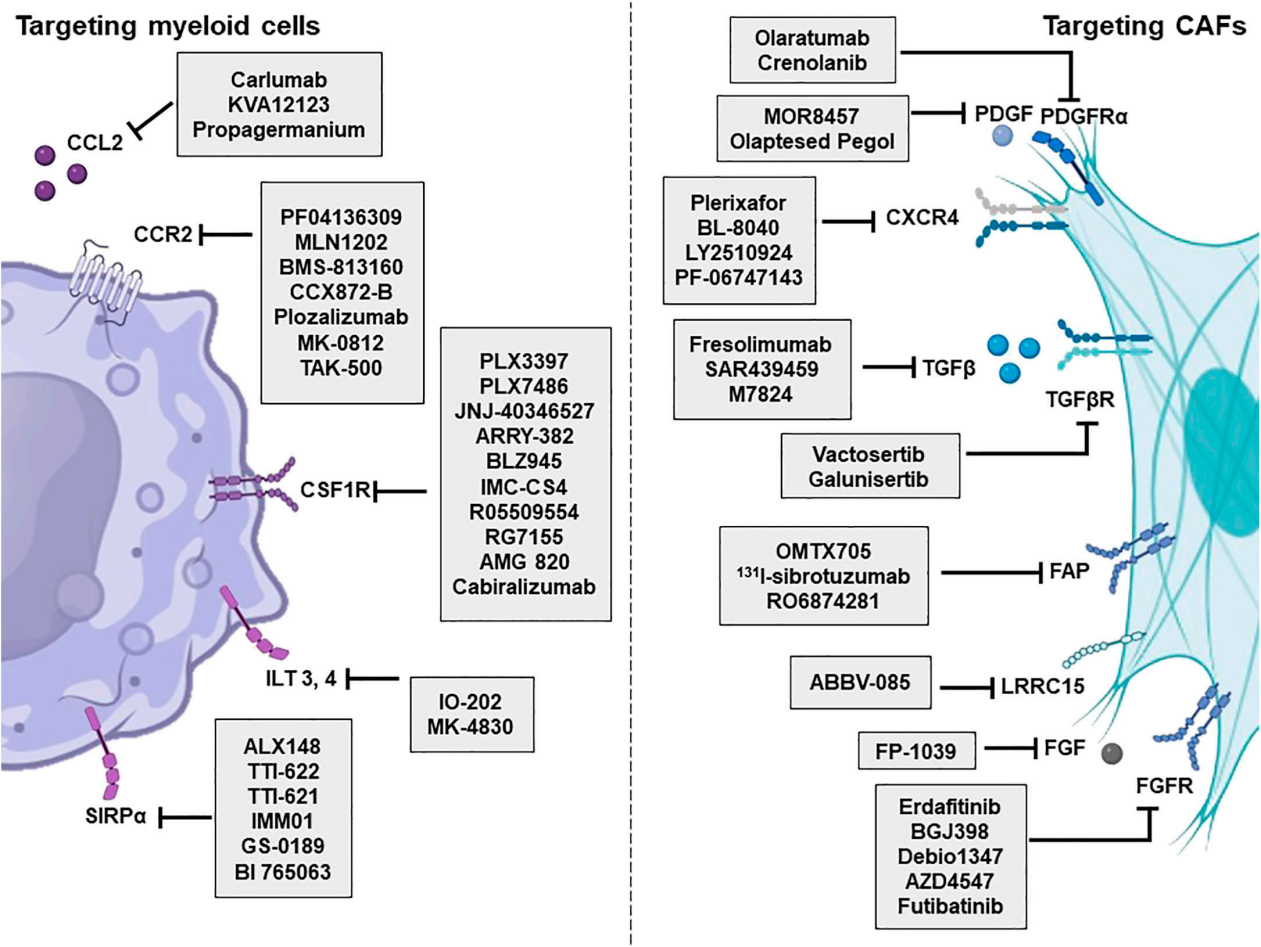
Therapeutic strategies targeting CAFs and myeloid cells in clinical development. Small molecules, monoclonal antibodies, fusion protein, antibody conjugates, and tyrosine kinase inhibitors can be used for therapeutic drugs targeting CAFs and myeloid cells.

The combinatorial targeting of CAFs and myeloid cells with other immunotherapy modalities offers a synergistic approach to enhance antitumor immune responses and overcome therapeutic resistance [40]. For instance, combining CAF-targeted therapies with immune checkpoint inhibitors can synergistically unleash cytotoxic T cell-mediated tumor eradication while simultaneously mitigating stromal-mediated immunosuppression [40,47]. Moreover, rational combinations of therapies targeting multiple components of the TME, including CAFs, myeloid cells, and angiogenic factors, may elicit durable antitumor responses and improve clinical outcomes [39,40]. Preclinical studies investigating these combination approaches are underway, offering hope for more effective and durable cancer therapies [48]. To effectively combat the intricate TME and its contribution to cancer progression, various therapeutic strategies targeting CAFs and myeloid cells are actively advancing in clinical settings (Fig. 3). Tables S3 and S4 illustrate the clinical progress of numerous drugs targeting these critical components of the TME.

### Existing hurdles in targeting CAFs and myeloid cells

Efforts to therapeutically target CAFs and myeloid cells encounter formidable challenges inherent to the complex and dynamic nature of the TME. First, CAFs exhibit substantial heterogeneity both within and between tumors, with distinct subsets exerting divergent effects on tumor behavior [36]. This heterogeneity poses challenges in identifying universally applicable therapeutic targets. Additionally, the dynamic crosstalk between cancer cells and stromal components often leads to compensatory mechanisms that undermine the efficacy of single-agent therapies [40,49].

Myeloid cells within the TME display remarkable plasticity, responding adaptively to microenvironmental cues. Their phenotypic and functional diversity complicates therapeutic interventions aimed at selectively targeting immunosuppressive subsets while sparing antitumor effectors [41]. Additionally, the infiltration of myeloid cells into tumors is often orchestrated by chemotactic gradients, posing challenges in disrupting these recruitment mechanisms without perturbing physiological myeloid cell trafficking [43]. Furthermore, the intricate interplay between CAFs, myeloid cells, and other TME constituents underscores the need for holistic therapeutic strategies that consider the multifaceted interactions within the tumor ecosystem [48].

### Future directions for enhancing treatments targeting CAFs and myeloid cells

The complex challenges of targeting CAFs and myeloid cells in cancer immunotherapy call for innovative research directions. To address these obstacles and propose effective future research paths, several advanced methodologies are being explored. For instance, single-cell RNA sequencing (scRNA-seq) and other single-cell analysis techniques are at the forefront, offering a detailed understanding of cell heterogeneity through comprehensive gene expression profiles at the individual cell level [40]. Proteomics techniques provide an in-depth analysis of protein expressions and interactions, enabling researchers to understand the functional implications of the proteins encoded by the genes identified in single-cell analyses [39]. Complementary methods, such as CyTOF, multiplex flow cytometry, and multiplex immunostaining, offer additional validation by allowing for the simultaneous measurement of multiple parameters across thousands of cells, thereby corroborating single-cell analysis results [50]. Intravital imaging provides real-time visualization of cell interactions and behaviors within the living TME, offering dynamic insights crucial for developing effective interventions [42,51]. Collectively, these advanced approaches are set to improve our understanding and capability to effectively target CAFs and myeloid cells, paving the way for more precise and effective cancer immunotherapies.

Developing new molecules that can target CAFs or myeloid cells in the TME, as well as promoting the migration of proinflammatory immune cells like T cells into the TME, could usher in a new era of TME modulation treatment. It is expected to make synergistic effect with CAR T cell therapy while treating solid tumor, overcoming the limitations of TME’s suppressive nature and tumor infiltration. Adhesion molecules and the low binding pseudoantigens could be a clue for this approach.

## AI-Aided Identification of Neoantigens and T Cell Epitopes

To accelerate the development of cancer immunotherapies, various computational methods have been developed. In this section, we will focus on the recent advances in MHC I/II interaction prediction and T cell activation prediction.

### Database for model training

The most used database for training machine learning (ML)/artificial intelligence (AI) models for cancer immunotherapy is the Immune Epitope Database and Analysis Resource (IEDB) [52]. IEDB is a freely available expert-curated database containing the information on experimentally verified immune epitopes. The database provides the information on epitope sequences, 3D structures, disease association, peptide–MHC (pMHC) binding affinities, and T cell/B cell immune responses obtained from biochemical assay and mass spectrometry experiments. Additionally, IEDB provides epitope analysis tools to identify potential epitopes from sequences. The motif analysis tools recognize shared sequence motifs from epitope sequences. As of March 2023, IEDB provides more than 1.6 million epitopes, 1.9 million T cell/B cell assays, and 4.7 million MHC ligand assay data entries. Other public databases are The Cancer Immune Atlas (TCIA) [53] and TANTIGEN [54]. TCIA provides cancer immunogenic information on neoantigens, leukocyte fraction, and immunogenicity. For T cell response data, PIRD [55], VDJdb [56], McPAS-TCR [57], and 10X [58] are commonly used.

### pMHC interaction prediction methods

Majority of pMHC interaction prediction models predict pMHC I interaction due to a larger number of experimental data and small variations of peptide length. The peptide binding groove of MHC I has closed ends, allowing only short peptides consisting of 8 to 10 amino acids to bind. Overall, pMHC I prediction models have higher accuracy than pMHC II prediction models.

One of the widely used pMHC I predictors is NetMHC, which is based on artificial neural network and trained with binding affinity measurement data [59,60]. Afterward, NetMHC has been improved in terms of both model architecture and the sizes of dataset [59–61]. The latest version, NetMHCpan-4.1, is trained with 850,000 quantitative data and consists of the ensemble of multi-layer perceptron (MLP) models. The model predicts pMHC I binding with a positive predictive value (PPV) (precision) of 0.829.

Kim et al. [62–64] developed DeepNeo predicting pMHC I and MHC II binding using a deep neural network algorithm. pMHC binding assay data are collected from IEDB, IMMA2 [65], and MHCBN [66]. The final dataset consists of 125,300 and 74,838 data points for MHC I and MHC II. The input of the model is a 2D interaction matrix between all amino acids of a given MHC molecule and a peptide filled with Cα–Cα distance-based amino acid preference values. The input matrix is processed with convolutional neural network (CNN) layers.

MHCflurry-2.0 is another pMHC I predictor, which consists of 2 modules: binding affinity predictor and antigen processing predictor [67]. The binding affinity predictor is a pan-allele pMHC I interaction predictor using the ensemble of MLP models. The antigen processing predictor distinguishes mass spectrometry hits from decoys and is composed of the one-dimensional (1D) CNN layers. MHCflurry-2.0 provides a combined presentation score, integrating the binding affinity and antigen processing predictions using a logistic regression model. MixMHCpred [68,69] predicts pMHC I interaction using the framework of a mixture model, an ML algorithm to detect subpopulations from a whole population. The latest version of MixMHCpred is trained with 384,070 peptides from 119 human leukocyte antigen-I (HLA-I) alleles [69].

pMHC II prediction methods have been less studied due to fewer number of available experimental data and the longer and larger variations of peptide lengths, ranging from 13 to 25. Many pMHC I prediction methods have been extended to predict pMHC II binding. Similar to NetMHCpan, NetMHCIIpan also employed an artificial neural network algorithm and is trained with extensive dataset of over 500,000 measurements of binding affinity and mass spectrometry [60]. MixMHC2pred is also a widely used pan-allele pMHC II binding predictor [70]. It is based on available mass spectrometry-based MHC-II peptidomics datasets consisting of 615,361 unique peptides. It consists of 2 neural network models. The first model predicts binding specificity based on the MHC II peptide binding site sequences. The second accepts a peptide sequence and the embedding vector from the first model as inputs and predicts their affinity. DeepNeo is also expanded to predict the pMHC II binding prediction, DeepNeo-v2. Compared to its first version, DeepNeo-v2 is trained with larger dataset [63].

### Peptide–TCR interaction prediction

The third category of neoantigen prediction methods is predicting T cell activity. NetTCR-2.0 is trained with 9,204 unique CDR3β sequences from IEDB [71]. NetTCR-2.0 consists of 1D-CNN layers. The CDR3α and CDR3β regions of the TCR amino acid sequences and a peptide sequence are used as inputs of the model. Each sequence is processed with separate 1D-CNN blocks, and resulting vectors are concatenated and further processed with an MLP block to predict binding probability. DeepNeo-v2 [63], a CNN-based MHC interaction predictor, also predicts T cell activation. Additionally, DeepTCR [72] and ImRex [73] also predict peptide–TCR interaction using the CNN architecture. epiTCR [74] is a TCR–peptide binding predictor based on the random forest algorithm [75] trained with more than >3 million TCR–peptide pair data from 5 public databases: IEDB [52], PIRD [55], VDJdb [56], McPAS-TCR [57], and 10X [58]. epiTCR used the TCR CDR3β sequences and neoantigen sequences as inputs. In addition to these methods, ATM-TCR [76] uses an attention-based neural network. ERGO-I [77] and pTMnet [78] are developed using the long short-term memory architecture.

Recently, due to the development of AlphaFold [79], a highly accurate protein 3D prediction model, structure-based prediction methods are emerging. The specialized version of AlphaFold that generates 3D models of TCR:pMHC interactions is developed [80]. The model discriminates correct from incorrect peptide epitopes with substantial accuracy. TCRmodel2 generates the complex structures of TCR-pMHC molecules based on the modified version of AlphaFold [81]. The methods reviewed in this section are listed in Table.

**Table.** List of AI/ML methods in immunotherapy

Name	Target	Algorithm	Training sets
NetMHCPan-4.1	MHC I	MLP	208,039 affinity values and 665,492 peptides
NetMHCIIpan-4.0	MHC II	MLP	108,959 affinity values and 381,066 peptides
MHCflurry-2.0	MHC I	CNN	219,596 affinity values and 493,473 peptides
MixMHCpred2.2	MHC I	Mixture model	384,070 interaction data and 258,814 peptides
MixMHC2pred	MHC II	Mixture model	627,013 peptides
DeepNeo-v2	MHC I MHC II TCR-pMHC	CNN	340,987 affinity values for MHC I 106,908 affinity values for MHC II 19,149 immunogenicity data for MHC I 12,913 immunogenicity data for MHC II
NetTCR-2.0	TCR-pMHC	CNN	9,204 CDR3β-peptide pairs and 4,593 CDR3α-/β-peptide pairs
pTMnet	TCR-pMHC	Long short-term memory	172,422 affinity values and 32,607 TCR-pMHC pairs
epiTCR	TCR-pMHC	Random Forest	3,255,086 CDR3β-epitope combinations
TCRdock	TCR-pMHC	AlphaFold	93 human ternary structures
TCRmodel2	TCR-pMHC	AlphaFold	48 ternary structures

Computational methods are promising tools when designing antibodies, providing the activity of immune responses against neoantigens that are not known yet. Especially, it can save our time and efforts to develop multi-specific antibodies. Visualizing the interaction between antibodies and other proteins, we can easily modulate the affinity in the AlphaFold system by changing one amino acid in the antibody sequence. The 3D model enables us to predict whether the antibody works well, based on the distance, steric hindrance, and affinity before the experiment. AI-developed therapeutic antibodies would give us great opportunities to treat various cancers in the future.

## Autologous Cancer Vaccines as Next-Generation Personalized Immunotherapy

Cancer vaccines represent a promising approach in the field of immunotherapy, and they can be broadly classified into 2 categories: preventive and therapeutic vaccines (Table S5). One successful example of a preventive cancer vaccine is the human papillomavirus (HPV) vaccine. The HPV vaccine works by stimulating the immune system to produce antibodies against specific strains of the virus, thereby preventing HPV infection and subsequent development of associated cancers [82]. The introduction of HPV vaccines, such as Gardasil and Cervarix, has led to a significant reduction in HPV infections and related diseases in vaccinated populations [83]. Clinical trials have demonstrated the efficacy of these vaccines in reducing the incidence of cervical cancer [84,85]. The success of HPV vaccines highlights the potential of preventive cancer vaccines in reducing the burden of cancer on a population level and underscores the importance of vaccination as a primary prevention strategy against certain cancers [86]. Unlike cancers induced by oncogenic viruses, it is very difficult to develop preventive vaccines against most cancers due to the lack of common cancer-specific antigens [87]. Moreover, while viral infection can be efficiently neutralized by antibodies generated by vaccination, generation of antibodies to cancer-specific antigens does not necessarily confer cancer prevention.

To develop a therapeutic vaccine to a certain cancer, we first need to define cancer neoantigen(s) that can be recognized by host’s immune system. However, identifying cancer neoantigens is challenging due to several reasons. First, neoantigens are unique to individual tumors, arising from somatic mutations, making them highly heterogeneous and difficult to predict [88]. Second, the mutational landscape of tumors can be complex, with a wide range of mutations occurring across the genome, further complicating the identification of neoantigens [89]. Third, distinguishing between neoantigens and self-antigens or nonimmunogenic mutations requires sophisticated bioinformatics tools and experimental validation, which can be time-consuming and resource-intensive [90]. Fourth, not all peptides that can be loaded onto MHC molecules trigger T cell activation, and the lack of standardized neoantigen prediction and validation further complicates the process [91]. These factors collectively contribute to the difficulty in accurately identifying cancer neoantigens, presenting a significant hurdle in the development of neoantigen-based immunotherapies, particularly therapeutic cancer vaccines.

### Cell-based cancer vaccines

To overcome the difficulties in defining neoantigens, investigators have studied the use of cancer cells themselves as vaccine sources [92]. These vaccines are developed by harvesting a patient’s own cancer cells, manipulating them to be immunogenic, termed autologous cancer vaccines. For instance, it has been shown that tumor lysate-pulsed dendritic cells and tumor antigen-pulsed dendritic cells can induce a prolonged survival and a reduced T regulatory (T_reg_) ratio in glioblastoma [93]. These types of dendritic cell-based cancer vaccine approaches have led to the development of sipuleucel-T (Provenge), a dendritic cell-based vaccine approved by U.S. FDA for the treatment of prostate carcinoma [94].

### Engineered whole cancer cell-based cancer vaccines

**Fig. 4.**
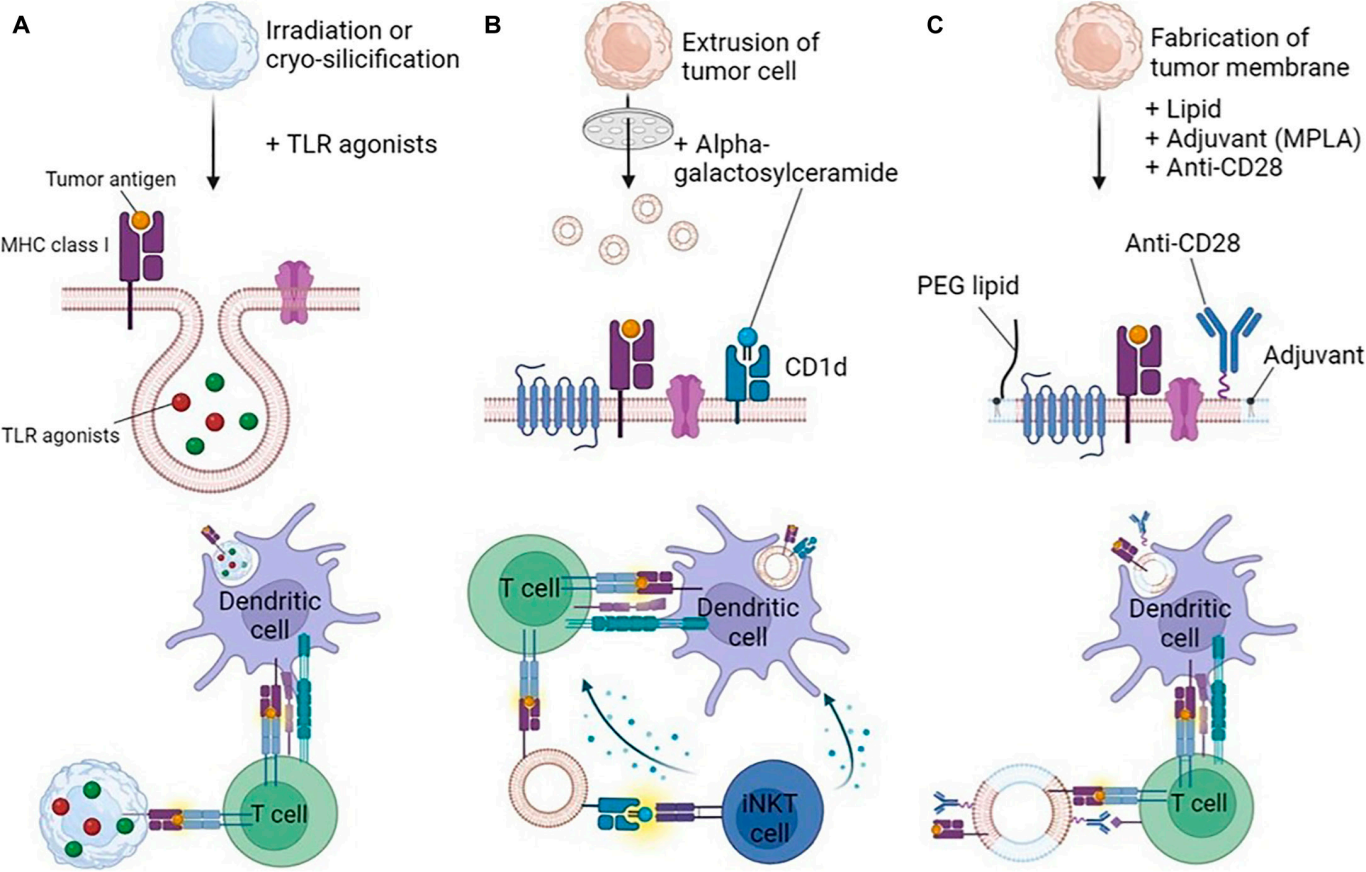
Next-generation platforms for autologous cancer vaccine. (A) Vaccination utilizing silicified and surface-modified tumor cells with TLR agonists enhances dendritic cell uptake and subsequent activation of T cells. (B) Vaccination with nanovesicles derived from tumor cells loaded with iNKT cell ligands activates both iNKT cells and antigen-specific T cell responses. (C) Vaccination using engineered nanovesicles conjugated with anti-CD28 antibodies facilitates direct interaction with T cells, thereby enhancing immune responses.

In addition to dendritic cell-based vaccines, recent studies have shown promising efficacy in reducing tumor burden by using autologous whole-cell cancer vaccines. The use of whole cancer cells as vaccine sources has advantages in that it poses a whole spectrum of neoantigens, obviating the challenges in defining specific neoantigens for vaccine development. To achieve vaccine efficacy, the whole-cell autologous cancer vaccines should preserve neoantigens. In addition, the vaccines should be engineered either to express adjuvants such as PAMPs that can efficiently activate dendritic cells that endocytose the cancer cells or to act as antigen-presenting cells that directly activate T cells specific for the neoantigens [95]. The use of whole cancer cells has been investigated as a personalized cancer vaccine [96–98]. For instance, Guo et al. [99] have recently developed a novel autologous cancer vaccine platform. They utilized a method called “cryo-silicification” that enables preservation of cell integrity and the biofunctionality of cellular proteins including neoantigens (Fig. 4A). They further engineered the cryo-silicified cancer cells to adsorb adjuvants such as ligands for Toll-like receptor 4 (TLR4) and TLR9. This strategy was shown to create autologous cancer vaccine within 24 hours without time-consuming and technically challenging manipulation. Vaccination with these cryosilicified autologous cancer cells showed a profound therapeutic efficacy, which was superior to vaccination with irradiated cancer cells or adjuvant alone. More importantly, the vaccination induced tumor-specific T_H_1 and cytotoxic T cells and was synergized with chemotherapy such as cisplatin. Interestingly, these autologous vaccines can be dehydrated and stored at room temperature without losing vaccine efficacy upon rehydration. Similarly, autologous whole cancer cell vaccines composed of irradiated cancer cells pulsed with TLR agonists and anti-CD40 were shown to induce a strong anticancer immunity and prolonged survival of tumor-bearing mice in a way dependent on T cells [100]. Therefore, the use of engineered whole cancer cells obtained from ascites or from surgical procedure may offer a promising platform for the development of individualized cancer vaccines.

### Whole cancer cell-based nanovesicle vaccines

The use of cancer-derived nanovesicles may offer another effective platform for the development of autologous cancer vaccines. Cancer-derived nanovesicles can be spontaneously produced by cancer cells or can be obtained by extrusion using nanoporous polycarbonate membranes [101]. If these autologous cancer-derived nanovesicles can trigger T cell immunity against the neoantigenic epitope, it can be developed as a novel platform of cancer vaccine that does not require to define individual neoantigens and HLA types of cancer patients. However, the poor immunogenicity of cancer-derived nanovesicles makes them inappropriate unless further formulated to enhance immunogenicity, limiting the vaccine’s effectiveness.

To overcome such hurdles, recent studies have developed intriguing formulation strategies. For instance, in animal models of acute myeloid leukemia (AML), an invariant natural killer T (iNKT) cell-induced activation was used to make the nanovesicles immunogenic. iNKT cells recognize glycolipid antigens presented on the MHC I-like molecule CD1d, and most cells of hematopoietic origin express CD1d [102]. Cancer cells of hematologic malignancy, such as AML and lymphoma, thus also express CD1d and can activate iNKT cells if they present iNKT ligand such as α-galactosylceramide (αGC) onto CD1d. αGC, particularly cell-associated forms, is a well-known trigger of broad activation of immune cells, including NK cells, dendritic cells, and T cells, in various animal models of cancer [103–105]. When cancer cell-derived nanovesicles were formulated to load αGC onto their surface CD1d, they efficiently activated iNKT cells. More importantly, such formulated cancer-derived nanovesicles were shown to induce CD8^+^ T cells specific to cancer antigens, and established long-term memory T cell responses in vivo (Fig. 4B). Freshly isolated human AMLs also express CD1D regardless of disease status [106]. Chemotherapy or targeted therapy is the standard treatment for AML patients in the clinic, and it is noteworthy that vaccination with the iNKT ligand-loaded autologous AML nanovesicles synergistically inhibited the growth of AML cancer cells in animal models. Thus, this type of therapeutic cancer vaccine can be further developed to prevent recurrence of hematologic malignancies after standard treatments in humans.

In addition to extrusion-mediated generation of cancer cell-derived nanovesicles, recent studies have developed surrogate cancer antigen-presenting nanoparticles by fusing cancer cell membranes presenting neoantigenic epitope on MHC I and liposome [107]. The nanoparticles can be further formulated to contain adjuvants, such as MPLA, and T cell stimulators, such as anti-CD28, in order to improve immunogenicity and T cell-stimulatory function (Fig. 4C). As it utilizes cancer cell membrane as a source of cancer neoantigens, it also does not need to define epitope and corresponding HLA for personalized vaccination. Through surface MHC I + neoantigenic epitopes in combination with anti-CD28, it can directly stimulate CD8 T cells specific for cancer neoantigens. After engulfing into dendritic cells, it can activate them through adjuvants, making them efficient inducer of cancer-specific T cells after cross-presentation of the neoantigens.

The use of cancer cells themselves or nanovesicles/membranes is fascinating in that it avoids the need to identify tumor antigens for preparing personalized cancer vaccine. Since it targets to expand cancer-specific T cells by providing “signal 1” in vivo, this strategy is expected to synergize with immune checkpoint blockers that target to block co-inhibitory molecules (signal 2). In animal model systems, these approaches showed minimal toxicity in vivo. Future studies addressing long-term safety and careful dose titration will be needed to avoid vaccine-induced adverse effects including cytokine storm or hyperactivity of T cells.

## mRNA Vaccines for Cancer Treatment

In 2020, the SARS-CoV-2 vaccines mRNA-1273 [108] and BNT162b2 [109] marked a historic achievement as the first FDA-approved mRNA vaccines. As mRNA is a noninfectious platform that does not integrate into the genome, it ensures a safety profile free of infection or insertional mutagenesis issues [110]. Moreover, the cell-free and straightforward manufacturing process for mRNA vaccines ensures a swift, cost-effective, and scalable production. Over the past couple of decades, concerns related to undue immunogenicity and in vivo degradation, potentially leading to poor translatability, have been effectively addressed.

**Fig. 5.**
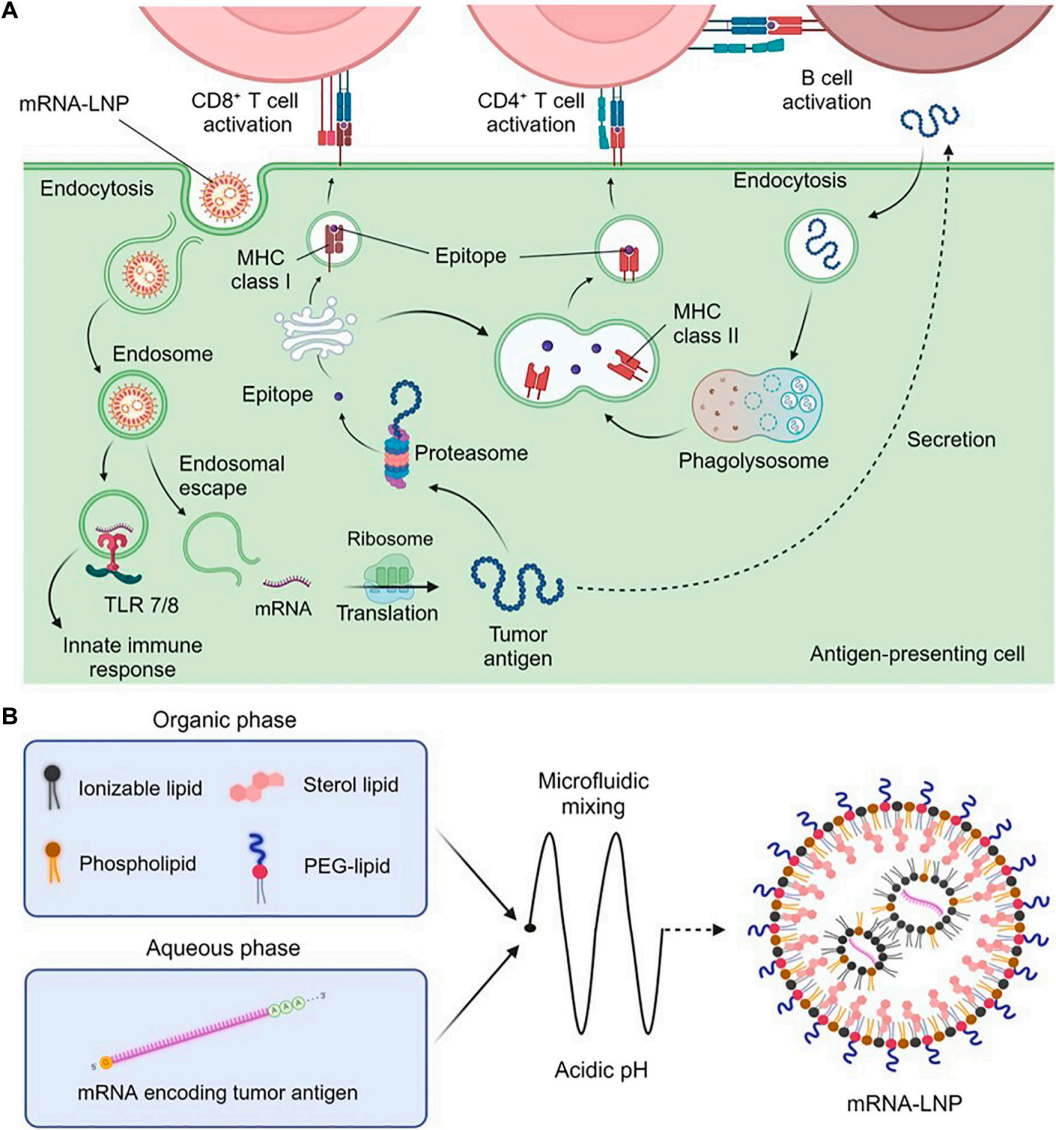
Preparation and therapeutic mechanism of mRNA-LNP cancer vaccines. (A) Pathway of activation for T cells and B cells specific to epitopes translated from mRNA delivered with LNPs. (B) Illustration of the preparation of an mRNA-LNP vaccine. mRNA encoding a tumor antigen is encapsulated by 4 types of lipids. Protonated ionizable lipids envelop negatively charged mRNAs at acidic pH.

The mode of action of mRNA vaccines involves several key steps (Fig. 5A). Once the mRNA is delivered to the cytosol of antigen-presenting cells, it undergoes translation, and the produced protein functions as an antigen. The resulting protein is either secreted or loaded onto an MHC I molecule. The MHC I immunopeptidome directly primes CD8^+^ T cells via antigen-presenting cells and activates antigen specifically. Meanwhile, the secreted protein is endocytosed by other antigen-presenting cells and presented on MHC II molecules. CD4^+^ T cells are stimulated by epitopes presented on MHC II molecules. Subsequently, CD4^+^ T cells encounter antigen-stimulated B cells at the T–B boundary in the B cell follicle and activate B cells. After germinal center reaction, B cells differentiate into plasma cells and produce antibodies against the antigen. Later, these cells contribute to the clearance of the antigen-displaying cancer cells [111,112].

### Nucleoside-modified mRNA vaccines

While it is challenging to meticulously control every step of the pathway, incorporating modified nucleosides such as N1-methylpseudouridine (m1ψ), 5-methoxyuridine (5moU), and 5-methylcytidine (m5C) into the transcript can enhance translation efficiency and reduce the innate immunogenicity of mRNA vaccines [108]. Reduced immune sensing of mRNA is advantageous for creating an anti-inflammatory environment [113], but it requires adjuvants when a robust immune response is necessary [114–116]. Although m1ψ is the most widely used, 5moU and m5C outperform it in the case of self-amplifying mRNA [117]. Therefore, it is critical to choose the optimal modified nucleoside based on the specific application.

Recently, m1ψ has encountered a new issue. Mulroney et al. [118] have observed that m1ψ may increase +1 ribosomal frameshifting, leading to the production of unintended proteins. However, as of now, there is no evidence suggesting that frameshifts compromise the safety of vaccines, and it is anticipated that frameshifting can be mitigated through codon optimization. For instance, using UUC instead of UUU to encode phenylalanine may be helpful, as frameshifting arises from ribosome stalling induced by m1ψ during in vitro transcribed mRNA translation. Further studies are needed to thoroughly understand this phenomenon and appropriately address this novel obstacle.

### Lipid nanoparticle–mRNA vaccines

Although mRNA has been a promising tool in the field of biology for an extended period, the substantial success of mRNA vaccines is largely attributed to the sophisticated carrier, lipid nanoparticle (LNP). Conventional LNPs comprise 4 types of lipids: ionizable lipid, phospholipid, sterol lipid, and polyethylene glycol (PEG)-lipid (Fig. 5B). Ionizable lipids, crucial for enveloping mRNA, undergo protonation and become positively charged at low pH. This feature enables them to encapsulate negatively charged mRNA in an acidic buffer, destabilizing endosomes after endocytosis, and subsequently facilitating the translocation of mRNA to the cytosol for translation. Phospholipids and sterol lipids are integrated into LNPs, serving as structural components that aid in cellular uptake, stabilize lipid bilayers, and modulate membrane fluidity [119]. Finally, PEG-lipid determines the particle size, reduces clearance by the immune system, and extends in vivo half-life. PEG-lipids in LNPs may induce pseudoallergies through complement activation and result in the production of anti-PEG antibodies [120]. Meanwhile, PEG-lipid can function as a bridge between the particle and targeting moieties [121]. A number of studies have explored the delivery of LNPs to specific cells using targeting moieties tethered to LNPs [122] or targeting specific tissues by modifying lipid composition [123]. The LNP itself is not inert and may elicit an immune response as a “foreign substance”, serving as adjuvants to enhance vaccine efficacy. Due to the modularity of the nanoparticle, the expression kinetics of mRNA delivered by LNPs can be further optimized to adjust the location, magnitude, and duration of protein expression, ultimately enhancing vaccine efficacy.

While vaccines generally aim for disease prophylaxis, mRNA plays a therapeutic role in many cancer vaccines. Despite this distinction, the underlying mechanism is similar. Much like how SARS-CoV-2 vaccines trained the immune systems to recognize the viral spike protein, cancer vaccines aim to introduce proteins presented on cancer cells. The critical step in constructing mRNA sequences is the careful selection of the antigen repertoire; however, a significant hurdle arises as there is often no explicit target. In cases where antigens are predefined, especially when expressed in the same cancer type in a considerable number of patients, the use of TSAs or TAAs may be an option [87]. A liposomal mRNA vaccine targeting 4 TAAs demonstrated the capability to elicit and sustain immune responses, reducing tolerance against the antigens in patients with melanoma [124]. Although this study showcased the practicality of using nonmutated and shared TAAs as mRNA vaccine targets, the low-level expression of TAAs in normal tissue often leads to central thymic tolerance [125] and on-target off-tumor toxicity. Moreover, there are a far more cases where antigens shared among cancer patients are absent.

### Personalized mRNA Vaccines

Fortunately, the identification of neoantigens has become feasible alongside the advancement of high-throughput gene sequencing and HLA typing [126]. Despite the high cost and labor-intensive process of iteratively determining optimal epitopes, numerous clinical trials utilizing personalized mRNA vaccines have been conducted. The inaugural in-human application of an mRNA cancer vaccine targeting neoantigens in 2017 involved the administration of 2 synthetic pentatope RNAs, constructed from 10 selected mutations per patient, leading to the successful priming of neo-epitope-specific T cells and sustained progression-free survival in melanoma [127]. Since 2020, several studies on neoepitope cancer vaccines have utilized LNP-formulated mRNAs. In one study, predicted neoepitopes and driver gene mutations were concatenated into a single mRNA construct and administered to patients with gastrointestinal cancer, resulting in the elicitation of KRAS G12D-specific T cells [128]. BioNTech and Moderna are also currently conducting clinical trials of BNT-122 and mRNA-4157, respectively. BNT-122, an mRNA-lipoplex, contains up to 20 personalized neoepitopes. The lipoplex for BNT-122 consists of cationic lipids, phospholipids, and cholesterol. BNT-122 was co-administered with atezolizumab. This combination expanded T cells that persisted for up to 2 years, showcasing enduring efficacy against mFOLFIRINOX in pancreatic cancer [129]. mRNA-4157, on the other hand, targets melanoma in combination with pembrolizumab. In a randomized phase 2b study, mRNA-4157 managed to lengthen recurrence-free survival in patients with resected high-risk melanoma when administered with adjuvant [130].

Beyond simply manufacturing of mRNAs encoding tumor antigens and encapsulating them in LNPs, trials have sought to enhance therapeutic efficacy. For instance, mRNA vaccines have been combined with adoptive cell therapy to boost the effectiveness of CAR T cells in solid tumors [131]. Additionally, a sequence encoding RNA polymerase has been introduced to antigen-encoding mRNA to enable self-amplification of antigen expression, leading to increased and consistent T cell responses even with lower doses [132]. Given the recent dominance of mRNA vaccines in the vaccine industry, the potential for further enhancements in both mRNA and LNP remains limitless.

In addition to mRNA engineering, envelope engineering is one of the emerging trends in the vaccine research. Enveloped delivery vesicles (EDVs) that have antibodies on the surface are engineered for on-target specificity and further developed using the CRISPR-Cas9 system to knock out specific genes of the target cells [133]. Virus-like particles (VLPs) are also developed to increase infection efficiency by introducing Gag-Pol mutations [134]. In vivo editing of the brain using VLP has been successfully performed in the neonatal mouse model.

Engineering of LNP could be a novel enhancement technology to increase the efficiency of target-specific delivery of mRNA. CAR-coated LNP is an example. This CAR is constructed with scFv on the surface of the LNP membrane and mRNA stabilizing protein inside of the LNP. Target tissue- or organ-specific mRNA delivery is possible through scFv on the surface, and mRNA is transferred into the host cell while maintaining its stable structure. This strategy can be further improved using AI to find the optimal lipid composition for CAR coating.

## Concluding Remarks and Perspectives

The landscape of cancer immunotherapy is continually evolving, driven by innovative technologies and interdisciplinary collaborations. The strategies discussed in this review highlight the remarkable progress made in overcoming hurdles in cancer treatment. From multi-specific antibodies to AI-predicted neoantigens and mRNA vaccines, each approach offers unique opportunities to enhance the efficacy and personalization of immunotherapy. Despite these advancements, challenges remain, including treatment resistance and the complexities of the TME.

The FDA-approved CAR T therapy has recently been well used in cancer treatment, especially in blood cancers. With its remarkable success, the CAR T cell therapy market is growing rapidly. Many other CAR T therapies are in clinical stage with different target and structures. However, the side effects represented by CRS and neurotoxicity, and tumor heterogeneity limit their efficacy in other cancer types including solid tumors.

The use of modified mRNA and advanced delivery systems has made mRNA cancer vaccines a more promising platform [108]. In addition, AI-predicted neoepitopes, tailored to the unique mutations of an individual’s tumor, can be swiftly encoded in mRNA [96]. Moderna and BioNTech, leaders in mRNA vaccine development, are currently conducting clinical trials using these cutting-edge technologies [129,130]. AI models play a crucial role in accelerating the development of personalized mRNA vaccines. Their AI models identify the most probable neoantigens and encode them into mRNA vaccines. These vaccines, which contain multiple neoantigens, have been shown to induce strong immune responses resulting in tumor suppression. However, challenges remain, including the need for more effective adjuvants to enhance the immune response and ensuring the scalability and cost-effectiveness of manufacturing personalized vaccines. In addition, AI models still face limitations in predictive power, particularly for MHC II neoantigen and immune response prediction, due to the limited size of available experimental data.

If the 5 innovative technologies work synergistically, it will be possible to overcome the current limitations in cancer immunotherapy. CAR T cells with multi-specific antigen-binding regions, new molecules targeting CAFs or myeloid cells that induce the migration of T cells, AI-developed therapeutic antibodies, and CAR-coated LNP are constructive improvement strategies described above. Continued research efforts aimed at elucidating underlying mechanisms and identifying novel targets are essential for further advancing cancer immunotherapy.
