# Supplementary material for: Strategies to Overcome Hurdles in Cancer Immunotherapy
Source: Biomater Res. 2024 Sep 19;28:0080. doi: 10.34133/bmr.0080 (PMC11411167; doi:10.34133/bmr.0080)
Supplement: Supplementary 1 — Tables S1 to S5 [file bmr.0080.f1.zip › 2024 BMR cancer immunotherapy review supplementary material.docx]

Supplemental Materials

**Strategies to overcome hurdles in cancer immunotherapy**

Jihyun Kim^1,†^, Byung Joon Lee^2,†^, Sehoon Moon^1,†^, Hojeong Lee^5^, Juyong Lee^1,6,7*^, Byung‐Soo Kim^2,3,4,*^ Keehoon Jung^5,*^, Hyungseok Seo^1,*^, and Yeonseok Chung^1,*^

^1^ Research Institute for Pharmaceutical Sciences, College of Pharmacy,

^2^ Interdisciplinary Program for Bioengineering,

^3^ School of Chemical and Biological Engineering,

^4^ Institute of Chemical Processes, Institute of Engineering Research, and BioMAX, Seoul National University, Seoul, 08826, Republic of Korea

^5^ Department of Anatomy and Cell Biology, Department of Biomedical Sciences, Seoul National University College of Medicine, Seoul, 03080, Republic of Korea

^6^ Molecular Medicine and Biopharmaceutical Sciences, Graduate School of Convergence Science and Technology, Seoul National University, Seoul 08826, Republic of Korea

^7^ Arontier Co., Seoul 06735, Republic of Korea

^†^These authors equally contributed to this work.

^*^Correspondence: J.L (nicole23@snu.ac.kr), B.-S.K (byungskim@snu.ac.kr), K.J. ( keehoon.jung@snu.ac.kr), H.S. (h.seo@snu.ac.kr), Y.C.([yeonseok@snu.ac.kr](mailto:yeonseok@snu.ac.kr))

**Table S1. Multi-specific antibodies in cancer immunotherapy.**

| Mechanisms | Drug Name | Targets | Key Indications | Trade Name / ID |
| --- | --- | --- | --- | --- |
| FDA-approved | | | | |
| Multi-tumor antigen targeting | Amivantamab | EGFR/cMet | NSCLC | Rybrevant |
| T cell engager | Blinatumomab | CD19/CD3 | B-ALL | Blincyto |
|  | Tebentafusp | GP100/CD3 | UM | Kimmtrak |
|  | Teclistamab | BCMA/CD3 | MM | Tecvayli |
|  | Mosunetuzumab | CD20/CD3 | FL | Lunsumio |
|  | Epcoritamab | CD20/CD3 | DLBCL | Epkinly |
|  | Glofitamab | CD20/CD3 | DLBCL | Columvi |
|  | Tarlatamab | DLL3/CD3 | SCLC | Imdelltra |
| Phase III | | | | |
| Multi-tumor antigen targeting | Zanidatamab | HER2/HER2 | HER2+ cancer, GC | NCT06282575 |
| Multi-immune checkpoint targeting | Erfonrilimab | PD-L1/CTLA-4 | NSCLC, PDAC | NCT05149326 |
|  | Cadonilimab | PD-1/CTLA-4 | LANPC | NCT05587374 |
| T cell engager | Catumaxomab | EpCAM/CD3 | GC | NCT04222114 |
|  | Odronextamab | CD20/CD3 | NHL, FL | NCT06091254 |
|  | Talquetamab | GPRC5D/CD3 | MM | NCT06208150 |
| Drug/Cytokine or its receptor conjugates | Ivonescimab | PD-1/VEGF | NSCLC | NCT05899608 |
|  | Navicixizumab | DLL-4/VEGF | Ovarian cancer | NCT05043402 |
|  | Bintrafusp Alfa | PD-L1/TGFβ | NSCLC | NCT03631706 |
| Phase II | | | | |
| Multi-tumor antigen targeting | Zenocutuzumab | HER2/HER3 | Breast cancer | NCT03321981 |
|  | IMM0306 | CD20/CD47 | NHL | NCT05805943 |
| T cell engager | Flotetuzumab^T^ | CD123/CD3 | AML | NCT02152956 |
| NK cell engager | AFM13 | CD30/CD16A | CD30^+^ PTCL | NCT05883449 |
| Phase I | | | | |
| Multi-tumor antigen targeting | DT2219ARL* | CD19/CD22 | B-ALL | NCT02370160 |
|  | HPN217 | BCMA/HSA/CD3 | MM | NCT04184050 |
|  | HPN328* | DLL3/HSA/CD3 |  | NCT04471727 |
| T cell engager | SAR442257 | CD38/CD28/CD3 | MM, NHL | NCT04401020 |
|  | SAR443216^T^ | HER2/CD28/CD3 | HER2^+^ solid tumor | NCT05013554 |
|  | NM21-1480^T^ | PD-L1/4-1BB/HSA | NSCLC | NCT04442126 |
|  | GNC-038 | PD-L1/CD19/  CD3/CD137 | NHL | NCT05623982 |
| NK cell engager | SAR443579* | CD123/NKp46/CD16 | AML, MDS | NCT05086315 |
|  | DF1001* | HER2/NKG2D/CD16 | HER2^+^ solid tumor | NCT04143711 |
| Drug/Cytokine  conjugates | GTB-3550^T^ | CD33/IL-15/CD16 | AML | NCT03214666 |

B-ALL, B cell lymphoblastic leukemia; NSCLC, Non-small cell lung cancer; UM, Uveal melanoma; MM, Multiple melanoma; FL, Follicular lymphoma; DLBCL, Diffuse large B cell lymphoma; SCLC, small cell lung cancer; GC, Gastric cancer; PDAC, Pancreatic ductal adenocarcinoma; LANPC, Locoregionally-advanced nasopharyngeal carcinoma; NHL, Non-Hodgkin’s lymphoma; FL, Follicular lymphoma; AML, Acute myeloid leukemia; PTCL, Peripheral T cell lymphoma; MDS, Myelodysplasia.
*Phase I/II, ^T^Terminated.

**Table S2. Selected CAR-T therapy in clinical trials.**

| Name | Target | Phase | Key Indications | ID | Note |
| --- | --- | --- | --- | --- | --- |
| NKTR-255 | CD19 | II/III | NHL, DLBCL | NCT05664217 | IL-15R agonist fusion |
| CART2219.1 | CD19/CD22 | I/II | B-ALL | NCT05429905 | Bispecific |
| CT041 | CLDN18.2 | I/II | GC, PC | NCT04581473 | Autologous |
| CHM-2101 | Cadherin17 | I/II | GC, CC | NCT06055439 | Autologous |
| CTO1681 | CD19 | I/II | DLBCL | NCT05905328 | CRS-reduced |
| ALLO-647 | CD52 | II | LBCL | NCT05714345 | Allogenic |
| BEAM-201 | CD7 | I/II | T-ALL, T-LL | NCT05885464 | Allogenic |
| ATHENA | CD19 | I/II | NHL | NCT06323525 | Power3 gene KO |

NHL, Non-Hodgkin’s lymphoma; DLBCL, Diffuse large B cell lymphoma; B-ALL, B cell acute lymphoblastic leukemia; GC, Gastric cancer; PC, Pancreatic cancer; CC, Colorectal cancer; LBCL, large B cell lymphoma; T-ALL, T cell acute lymphoblastic leukemia; T-LL, T cell lymphoblastic leukemia.

**Table S3. Clinical progress of drugs targeting myeloid cells.**

| **Target** | **Agent** | **Therapeutic category** | **Mechanism of action** |
| --- | --- | --- | --- |
| CCL2 | Carlumab | mAb | targets and neutralizes CCL2 |
| CCL2 | KVA12123 | mAb | blocks VISTA (V-domain Ig suppressor of T cell activation), a negative immune checkpoint |
| CCL2 | Propagermanium | Small molecule | reduces the production of CCL2 |
| CCR2 | PF04136309 | Small molecule | prevents the migration of monocytes and macrophages into the TME |
| CCR2 | MLN1202 | Small molecule | prevents the migration of monocytes and macrophages into the TME |
| CCR2 | BMS-813160 | Small molecule | prevents the migration of monocytes and macrophages into the TME |
| CCR2 | CCX872-B | Small molecule | prevents the migration of monocytes and macrophages into the TME |
| CCR2 | Plozalizumab | mAb | prevents the migration of monocytes and macrophages into the tumor microenvironment |
| CCR2 | MK-0812 | Small molecule | prevents the migration of monocytes and macrophages into the tumor microenvironment |
| CCR2 | TAK-500 | immune-stimulating antibody conjugate | prevents the migration of monocytes and macrophages into the tumor microenvironment |
| CSF1R | PLX3397 | Small molecule | reduces the survival and recruitment of TAMs |
| CSF1R | PLX7486 | Small molecule | reduces the survival and recruitment of TAMs |
| CSF1R | JNJ-40346527 | Small molecule | reduces the survival and recruitment of TAMs |
| CSF1R | ARRY-382 | Small molecule | reduces the survival and recruitment of TAMs |
| CSF1R | BLZ945 | Small molecule | reduces the survival and recruitment of TAMs |
| CSF1R | IMC-CS4 | mAb | reduces the survival and recruitment of TAMs |
| CSF1R | R05509554 | mAb | reduces the survival and recruitment of TAMs |
| CSF1R | RG7155 | mAb | reduces the survival and recruitment of TAMs |
| CSF1R | AMG 820 | mAb | reduces the survival and recruitment of TAMs |
| CSF1R | Cabiralizumab  (FPA008) | mAb | reduces the survival and recruitment of TAMs |
| CD47 | Hu5F9-G4 | mAb | enhances the phagocytosis of cancer cells by macrophages |
| CD47 | CC-90002 | mAb | enhances the phagocytosis of cancer cells by macrophages |
| SIRPα | ALX148(Evorpacept) | fusion protein | promotes the phagocytosis of tumor cells through Fcγ receptors by blocking the CD47-SIRPα interaction. |
| SIRPα | TTI-622 | SIRPα-Fc fusion protein | promotes the phagocytosis of tumor cells through Fcγ receptors by blocking the CD46-SIRPα interaction. |
| SIRPα | TTI-621 | SIRPα-Fc fusion protein | promotes the phagocytosis of tumor cells through Fcγ receptors by blocking the CD47-SIRPα interaction. |
| SIRPα | IMM01 | SIRPα-Fc fusion protein | promotes the phagocytosis of tumor cells through Fcγ receptors by blocking the CD48-SIRPα interaction. |
| SIRPα | GS-0189 | mAb | blocking the inhibitory signal from CD46 and promoting macrophage activation and phagocytosis |
| SIRPα | BI 765063(OSE-172) | mAb | blocking the inhibitory signal from CD47 and promoting macrophage activation and phagocytosis |
| ILT3 | IO-202 | mAb | inhibits ILT3’s immunosuppressive function |
| ILT4 | MK-4830 | mAb | blocks ILT4, a myeloid-specific immune checkpoint, thus preventing it from suppressing the immune response |
| Caspase 8 | Trabectedin | Small molecule | induces apoptosis in myeloid cells within the TME |
| CD40 | CP-870,893 | agonistic antibody | activates CD40 on antigen-presenting cells, including myeloid dendritic cells |
| CD40 | RO7009789 | agonistic antibody | activates CD40 on antigen-presenting cells, including myeloid dendritic cells |
| TLR7 | Imiquimod | Small molecule | activates myeloid dendritic cells and macrophages to produce pro-inflammatory cytokines |
| TLR7 | 85ZA | Small molecule | activates myeloid dendritic cells and macrophages to produce pro-inflammatory cytokines |
| TLR9 | IMO-2055 | Small molecule | stimulates myeloid dendritic cells to produce cytokines and enhance the immune response against tumors |
| TLR9 | CMP-001 | Small molecule | stimulates myeloid dendritic cells to produce cytokines and enhance the immune response against tumors |
| TLR9 | SD-101 | Small molecule | Stimulates myeloid dendritic cells to produce cytokines and enhance the immune response against tumors |
| IDO1 | Epacadostat | Small molecule | reduces the suppression exerted by MDSCs and other regulatory immune cells |
| PDE5 | Tadalafil | Small molecule | reduces the suppressive function of MDSCs |
| PDE5 | Sildenafil | Small molecule | reduces the suppressive function of MDSCs |
| COX-2 | Apricoxib | Small molecule | decreases the production of prostaglandin E2 (PGE1), which can inhibit the function of MDSCs |
| COX-2 | Celecoxib | Small molecule | decreases the production of prostaglandin E2 (PGE2), which can inhibit the function of MDSCs |
| C-kit, PDGFR, BCR-ABL | Imatinib | Tyrosine kinase inhibitor | reduces the recruitment of TAMs and other myeloid cells |
| C-kit | Masitinib | Tyrosine kinase inhibitor | reduces the recruitment of TAMs and other myeloid cells |
| C-kit, SRC, ABL | Dasatinib | Tyrosine kinase inhibitor | reduces the recruitment of TAMs and other myeloid cells |
| C-kit, FGFR, VEGFR | Dovitinib | Tyrosine kinase inhibitor | reduces the recruitment of TAMs and other myeloid cells |
| HER2 | CT-0508 | CAR-M | enhances antigen-dependent phagocytosis and pro-inflammatory M1 Polarization |

**Table S4. Clinical progress of drugs targeting CAFs.**

| **Target** | **Agent** | **Therapeutic category** | **Mechanism of action** |
| --- | --- | --- | --- |
| Hyaluronic acid | PEGPH20 | PEGylated enzyme | Enzymatic degradation of excessive stromal hyaluronan |
| CTGF | Pamrevlumab  (FG-3019) | Human mAb | Antagonizes the action of CTGF |
| LOXL2 | Simtuzumab  (GS-6624) | Human mAb | Blocks fibroblast recruitment and collagen cross-linking functions of LOXL2 |
| PDGF | MOR8457 | mAb | Inhibits PDGF-mediated signaling pathways |
| PDGF | Olaptesed Pegol (NOX-A12) | synthetic L-stereoisomer RNA oligonucleotide | Reduces the growth and survival of CAFs and disrupts their ability to remodel the extracellular matrix |
| PDGFRα/β | Crenolanib | small molecule | Reduces the growth and survival of CAFs and disrupts their ability to remodel the extracellular matrix |
| PDGFRα | Olaratumab | Human mAb | Antagonizes PDGFRα signaling |
| IGF1 and IGF2 | Xentuzumab  (BI 836845) or MEDI-573 | Human mAb | Inhibits IGF1-stimulated and IGF2-stimulated activation of IGFRs |
| IL-6 | Siltuximab | Chimeric monoclonal antibody | Blocks IL-6–IL-6R signaling |
| IL-6R | Tocilizumab | humanized monoclonal antibody | Blocks IL-6–IL-7R signaling |
| IL-6 and TGF-β | Pirfenidone | Antifibrotic agent | Inhibits fibroblast activity and disrupts the crosstalk between NSCLC cells and CAFs |
| TGFβ | SAR439459 | Small-molecule inhibitor | Alleviates the immunosuppressive effects of TGF-β in the tumor microenvironment by neutralizing TGF-β activity |
| TGFβ | Fresolimumab (GC1008) | Human mAb | Alleviates the immunosuppressive effects of TGF-β in the tumor microenvironment by neutralizing TGF-β activity |
| TGFβR I inhibitor | Vactosertib | Small-molecule inhibitor | Blocks TGFβ signaling and CAF activation |
| TGFβR I inhibitor | Galunisertib (LY2157299) | Small-molecule inhibitor | Blocks TGFβ signaling and CAF activation |
| TGF-β and PD-L1 | M7824 (Bintrafusp alfa) | Bifunctional fusion protein | Inhibits T-cell activation and TGF-β that promotes immunosuppression and fibrosis |
| FAP | OMTX705 | Antibody-drug conjugate (ADC) | Leads to cell cycle arrest and apoptosis of tumor cells while depleting the stroma and reducing tumor growth |
| FAP | ^131^I-sibrotuzumab | Radioactive isotope conjugated antibody | ^131^I-labeled FAP-targeting antibody uptake in FAP-positive tumors |
| FAP | RO6874281  (FAP-IL2v, RG7461) | Antibody moiety linked to cytokine (immunocytokine) | Binding to FAP and targeting IL2 (IL-2v) to FAP-expressing cells/tumors |
| Vitamin A metabolism | ATRA | Metabolite | CAF normalization and restrained tumor growth |
| Vitamin D receptor | Paricalcitol  (vitamin D analogue) | Small molecule | CAF normalization and improved chemotherapeutic efficacy |
| Hedgehog | Vismodegib  (GDC-0449) | Small molecule | Inhibition of HH–SMO signaling and tumor growth inhibition. Potential deactivation of CAFs |
| Hedgehog | LDE225 (Sonidegib) | Small molecule | Inhibition of SMO and HH signaling pathway |
| Hedgehog | Saridegib or patidegib (IPI-926) | Small molecule | Inhibition of HH–SMO signaling, stromal depletion and facilitated drug delivery |
| AXL | TP-0903 | Small molecule | Inhibition of AXL on cancer cells, CAFs, and myeloid cells |
| CXCR4 | Plerixafor(AMD3100) | Small molecule | Blocks CXCL12(SDF1)–CXCR4 interaction and immune modulation |
| CXCR4 | BL-8040 | Small molecule | CXCL12-CXCR4 inhibition and immune regulation |
| CXCR4 | LY2510924 | Peptide antagonist | CXCL12-CXCR4 inhibition and immune regulation |
| CXCR4 | PF-06747143 | mAb | CXCL12-CXCR4 inhibition and immune regulation |
| FAK | Defactinib (VS-6063, PF-04554878) | Small molecule | Restrains tumor growth through FAK inhibition |
| FGF | FP-1039 (GSK3052230) | fusion protein | inhibits FGF signaling and reduces tumor growth, angiogenesis, and stromal support |
| FGFR1-4 | Erdafitinib  (JNJ-42756493) | Small molecule | Inhibits FGFRs expressed by cancer cells and antagonizing the signaling induced by FGF released from CAFs |
| FGFR1-3 | BGJ398, Debio1347, AZD4547 or Futibatinib | Small molecule | Induces of CAF senescence or inactivation and inhibit tumor growth |
| VEGFR and FGFR tyrosine kinases | Brivanib  (BMS-582664) | Small-molecule inhibitor | Impairs the ability of CAFs to promote tumor growth and metastasis |
| PDGFR, BCR–ABL1 and KIT | Imatinib | Tyrosine kinase inhibitor | Inhibits PDGFR in CAFs and pericytes and inhibits tumor growth and angiogenesis |
| SRC and ABL1 | Dasatinib | ATP-competitive tyrosine kinase inhibitor | Inhibits cancer cells and CAFs |
| FGFR, PDGFR, and VEGFR | Nintedanib | Small molecule | Inhibits multiple receptor tyrosine kinases involved in the signaling pathways that promote fibroblast activation and angiogenesis |
| MMP9 | Andecaliximab  (GS-5745) | Recombinant chimeric IgG4 mAb | Inhibits MMP9 |
| MMPs | S-3304 | Small molecule | Inhibits MMPs and ECM degradation |
| LRRC15 | ABBV-085 | Antibody-drug conjugate | Delivers the cytotoxic agent monomethyl auristatin E (MMAE) directly to the tumor microenvironment |

**Table S5. Overview of the types and mechanisms of cancer vaccines in clinical trials.**

| Types of vaccines | | | Mechanisms |
| --- | --- | --- | --- |
| Preventive vaccines | | HPV vaccines | Contain virus-like particles that resemble the outer protein shell of the actual HPV virus, stimulating immune system to produce antibodies against the HPV type  - Product : Gardasil, Cervarix |
|  |  | HBV vaccines | Contain hepatitis B surface antigen (HBsAg) to prevent HBV infection and thereby reduces the risk of liver cancer  - Product : Engerix-B, Recombivax HB |
| Therapeutic vaccines | with known tumor antigens | Peptide/protein vaccines | Directly deliver specific tumor-associated antigens of fragments of these proteins to the immune system  - Product : HER2/neu peptide Nelipepimut-S (E75) |
|  |  | Dendritic cell vaccines | Use the patient’s own immune cells and activate them ex vivo against a prostate cancer-specific antigen  - Product: Provenge (Sipuleucel-T) |
|  |  | DNA/RNA vaccines | Deliver genetic instructions of tumor antigen production to induce both humoral and cellular immune responses  - Product : mRNA-4157 |
|  | with unknown tumor antigens | Tumor lysate vaccines | Use the cellular material derived from tumor cells that have been lysed. When combined with dendritic cells, these lysates can enhance the immune response |
|  |  | Autologous tumor vaccines | Use inactivated or modified tumor cells extracted from the patient to enhance their immunogenicity |
|  |  | Bystander vaccines | Activate the immune system in a way that targets not only the injected antigens but also nearby tumor cells that may express different antigens |

HPV, Human papillomavirus; HBV, Hepatitis B virus.
