# Supplementary material for: Strategies to Overcome Hurdles in Cancer Immunotherapy
Source: Biomater Res. 2024 Sep 19;28:0080. doi: 10.34133/bmr.0080 (PMC11411167; doi:10.34133/bmr.0080)
Supplement: Supplementary 1 — Tables S1 to S5 [file bmr.0080.f1.zip › BMR_COI_Form-combined.pdf]

Biomaterials Research
A SCIENCE PARTNER JOURNAL

Authorship Form and Statement of Conflicts of Interest

Author Name: Jihyun Kim

Manuscript Number: BMR-D-24-00200

Manuscript Title: Strategies to overcome hurdles in cancer immunotherapy

Each author must complete the following form prior to acceptance of their paper:

I. Authorship:

The authorship policies of *Biomaterials Research* follow those recommended by the report "On Being a Scientist", 3rd Edition, published by the US National Academy of Sciences (http://www.nap.edu/catalog/12192.html). In particular, we note that "just providing laboratory space for a project or furnishing a sample used in the research is not sufficient to be included as an author, although such contributions... may be recognized in a separate acknowledgment section."

In order to meet our requirements for authorship of a paper, you must have participated significantly in the reported research or writing of the paper. Please affirm that you meet these criteria by indicating your contribution to all of the following descriptions (from 0 to 100%):

Authorship Activity	Level of participation					
	0%	20%	40%	60%	80%	100%
Participated in the design and/or interpretation of the reported experiments or results.	●	○	○	○	○	○
Participated in the acquisition and/or analysis of data.<br>Indicate which data: Preventive and therapeutic cancer vaccine	○	○	○	○	○	●
Participated in drafting and/or revising the manuscript.	○	○	●	○	○	○
Primarily responsible for a particular, specialized role in the research (e.g. statistical analysis, crystallography, preparation of cell lines, etc)<br>Briefly state roles: drafting and revising the manuscript, figure4	○	○	○	●	○	○
Provided administrative, technical or supervisory support.	○	○	●	○	○	○

The senior author from each lab or group must answer this question: I have personally checked all the original data that was generated by my lab or group:

☑ Yes ☐ Not applicable; I am not the senior author or lab head.

If yes, these data are presented in these figures and tables (including the Supporting Online Material):

I have reviewed, or will review, the revised manuscript and approve of its submission to *Biomaterials Research* for publication.

☑ Yes ☐ No

If my university or institution has a separate publication license that applies to me (as required by Harvard, MIT, Open University, *et al.*) I have applied for a waiver.

☐ Yes ☑ There are no publication policies or restrictions limiting my ability to grant exclusive publication rights to The Korean Society for Biomaterials ("KSBM").

II. Data sharing:

I affirm that all data necessary for a reader of *Biomaterials Research* to understand and evaluate the conclusions of the paper will be archived in an approved database and made available to any reader.

☑ Yes ☐ No

III. Materials sharing:

I understand that after publication, all reasonable requests for materials and data must be fulfilled.

☑ Yes ☐ No

Biomaterials Research must be informed of any restrictions on sharing of materials [Materials Transfer Agreements (MTAs) or patents, for example] applying to materials used in the reported research.

☑ No, there are no MTAs

☐ Yes. Information on MTAs is described below.

IV. Conflict of Interest:

Biomaterials Research has a primary responsibility to its readers and to the public to provide in its pages clear and unbiased scientific results and analyses. We think that our readers should be informed of additional relationships of our authors that could pose a conflict of interest. Thus, for readers to evaluate the data and opinions presented in this journal, they must be informed of financial and other interests of our authors that may be at odds with unbiased presentation of data or analysis.

Therefore, *Biomaterials Research* believes that manuscripts should be accompanied by clear disclosures from all authors of their affiliations, funding sources, or financial holdings that might raise questions about possible sources of bias.
Disclosure is accomplished in three ways:

First, by a complete listing of the current institutional affiliations of the authors.

This list must include academic as well as corporate and other industrial affiliations. As the editors deem appropriate, items in this list will be included in the author affiliations printed in the manuscript. Please indicate below:
☑ _Yes, all my affiliations are listed on the title page of the paper.

Additional affiliations not on the title page are:

Second, through the acknowledgment of all financial contributions to the work being reported, including contributions "in kind." All funding sources will be listed in the published manuscript. Please indicate below:

☑ All my funding sources for this study are listed in the acknowledgement section of the paper.
☐ Additional funding sources not noted in the manuscript are:

Third, through the execution of a statement disclosing to the Editors all financial holdings, professional affiliations, advisory positions, board memberships, patent holdings and the like that might bear a relationship to the subject matter of the contribution. The Editors will determine whether the material disclosed to them should be published as part of the article.

The following are declarable relationships: **Financial**: Significant financial interest (equity holdings or stock options) in any corporate entity dealing with the material or the subject matter of this contribution. Please disclose the entity and the nature and amount of the holding:

- [x] None
- [ ] I have a financial relationship, as described below.

Management/Advisory affiliations: Within the last 3 years, status as an officer, a member of the Board, or a member of an Advisory Committee of any entity engaged in activity related to the subject matter of this contribution. Please disclose the nature of these relationships and the financial arrangements.

- [x] None
- [ ] I have a management/advisory relationship, as described below:

Paid Consulting: Within the last 3 years, receipt of consulting fees, honoraria, speaking fees, or expert testimony fees from entities that have a financial interest in the results and materials of this study. Please enumerate.

- [x] None
- [ ] I have a consulting relationship, as described below:

Patents: A planned, pending, or awarded patent on this work by any of the authors or their institutions. Please explain.

- [x] None
- [ ] I or my institution has a patent related to this work, as described below

Declaration: I declare that I have read *Biomaterials Research's* full Conflict of Interest Policy and have disclosed all declarable relationships as defined therein, if any.

This form was submitted on August 1st, 2024

Name Jihyun Kim

Signature

Biomaterials Research
A SCIENCE PARTNER JOURNAL

Authorship Form and Statement of Conflicts of Interest

Author Name: Byung Joon Lee

Manuscript Number: BMR-D-24-00200

Manuscript Title: Strategies to overcome hurdles in cancer immunotherapy

Each author must complete the following form prior to acceptance of their paper:

I. Authorship:

The authorship policies of *Biomaterials Research* follow those recommended by the report "On Being a Scientist", 3rd Edition, published by the US National Academy of Sciences (http://www.nap.edu/catalog/12192.html). In particular, we note that "just providing laboratory space for a project or furnishing a sample used in the research is not sufficient to be included as an author, although such contributions... may be recognized in a separate acknowledgment section."

In order to meet our requirements for authorship of a paper, you must have participated significantly in the reported research or writing of the paper. Please affirm that you meet these criteria by indicating your contribution to all of the following descriptions (from 0 to 100%):

Authorship Activity	Level of participation					
	0%	20%	40%	60%	80%	100%
Participated in the design and/or interpretation of the reported experiments or results.	○	●	○	○	○	○
Participated in the acquisition and/or analysis of data.<br>Indicate which data: mRNA cancer vaccine	○	●	○	○	○	○
Participated in drafting and/or revising the manuscript.	○	●	○	○	○	○
Primarily responsible for a particular, specialized role in the research (e.g. statistical analysis, crystallography, preparation of cell lines, etc)<br>Briefly state roles: drafting the manuscript	○	●	○	○	○	○
Provided administrative, technical or supervisory support.	○	●	○	○	○	○

The senior author from each lab or group must answer this question: I have personally checked all the original data that was generated by my lab or group:

☑ Yes ☐ Not applicable; I am not the senior author or lab head.

If yes, these data are presented in these figures and tables (including the Supporting Online Material):

I have reviewed, or will review, the revised manuscript and approve of its submission to *Biomaterials Research* for publication.

☑ Yes ☐ No

If my university or institution has a separate publication license that applies to me (as required by Harvard, MIT, Open University, *et al.*) I have applied for a waiver.

☐ Yes ☑ There are no publication policies or restrictions limiting my ability to grant exclusive publication rights to The Korean Society for Biomaterials ("KSBM").

II. Data sharing:

I affirm that all data necessary for a reader of *Biomaterials Research* to understand and evaluate the conclusions of the paper will be archived in an approved database and made available to any reader.

☑ Yes ☐ No

III. Materials sharing:

I understand that after publication, all reasonable requests for materials and data must be fulfilled.

☑ Yes ☐ No

Biomaterials Research must be informed of any restrictions on sharing of materials [Materials Transfer Agreements (MTAs) or patents, for example] applying to materials used in the reported research.

☑ No, there are no MTAs

☐ Yes. Information on MTAs is described below.

IV. Conflict of Interest:

Biomaterials Research has a primary responsibility to its readers and to the public to provide in its pages clear and unbiased scientific results and analyses. We think that our readers should be informed of additional relationships of our authors that could pose a conflict of interest. Thus, for readers to evaluate the data and opinions presented in this journal, they must be informed of financial and other interests of our authors that may be at odds with unbiased presentation of data or analysis.

Therefore, *Biomaterials Research* believes that manuscripts should be accompanied by clear disclosures from all authors of their affiliations, funding sources, or financial holdings that might raise questions about possible sources of bias.
Disclosure is accomplished in three ways:

First, by a complete listing of the current institutional affiliations of the authors.

This list must include academic as well as corporate and other industrial affiliations. As the editors deem appropriate, items in this list will be included in the author affiliations printed in the manuscript. Please indicate below:
☑ Yes, all my affiliations are listed on the title page of the paper.

Additional affiliations not on the title page are:

Second, through the acknowledgment of all financial contributions to the work being reported, including contributions "in kind." All funding sources will be listed in the published manuscript. Please indicate below:

☑ All my funding sources for this study are listed in the acknowledgement section of the paper.
☐ Additional funding sources not noted in the manuscript are:

Third, through the execution of a statement disclosing to the Editors all financial holdings, professional affiliations, advisory positions, board memberships, patent holdings and the like that might bear a relationship to the subject matter of the contribution. The Editors will determine whether the material disclosed to them should be published as part of the article.

The following are declarable relationships: **Financial**: Significant financial interest (equity holdings or stock options) in any corporate entity dealing with the material or the subject matter of this contribution. Please disclose the entity and the nature and amount of the holding:

- [x] None
- [ ] I have a financial relationship, as described below.

Management/Advisory affiliations: Within the last 3 years, status as an officer, a member of the Board, or a member of an Advisory Committee of any entity engaged in activity related to the subject matter of this contribution. Please disclose the nature of these relationships and the financial arrangements.

- [x] None
- [ ] I have a management/advisory relationship, as described below:

Paid Consulting: Within the last 3 years, receipt of consulting fees, honoraria, speaking fees, or expert testimony fees from entities that have a financial interest in the results and materials of this study. Please enumerate.

- [x] None
- [ ] I have a consulting relationship, as described below:

Patents: A planned, pending, or awarded patent on this work by any of the authors or their institutions. Please explain.

- [x] None
- [ ] I or my institution has a patent related to this work, as described below

Declaration: I declare that I have read *Biomaterials Research's* full Conflict of Interest Policy and have disclosed all declarable relationships as defined therein, if any.

This form was submitted on August 1st, 2024

Name Byung Joon Lee

Signature

Biomaterials Research
A SCIENCE PARTNER JOURNAL

Authorship Form and Statement of Conflicts of Interest

Author Name: Sehoon Moon

Manuscript Number: BMR-D-24-00200

Manuscript Title: Strategies to overcome hurdles in cancer immunotherapy

Each author must complete the following form prior to acceptance of their paper:

I. Authorship:

The authorship policies of *Biomaterials Research* follow those recommended by the report "On Being a Scientist", 3rd Edition, published by the US National Academy of Sciences (http://www.nap.edu/catalog/12192.html). In particular, we note that "just providing laboratory space for a project or furnishing a sample used in the research is not sufficient to be included as an author, although such contributions... may be recognized in a separate acknowledgment section."

In order to meet our requirements for authorship of a paper, you must have participated significantly in the reported research or writing of the paper. Please affirm that you meet these criteria by indicating your contribution to all of the following descriptions (from 0 to 100%):

**Authorship Activity**	**Level of participation**					
	0%	20%	40%	60%	80%	100%
Participated in the design and/or interpretation of the reported experiments or results.	●	○	○	○	○	○
Participated in the acquisition and/or analysis of data.<br>Indicate which data: Bispecific antibodies and CAR-T therapy in clinical development	○	●	○	○	○	○
Participated in drafting and/or revising the manuscript.	○	●	○	○	○	○
Primarily responsible for a particular, specialized role in the research (e.g. statistical analysis, crystallography, preparation of cell lines, etc)<br>Briefly state roles: revising the manuscript	○	○	○	●	○	○
Provided administrative, technical or supervisory support.	●	○	○	○	○	○

The senior author from each lab or group must answer this question: I have personally checked all the original data that was generated by my lab or group:

☑ Yes ☐ Not applicable; I am not the senior author or lab head.

If yes, these data are presented in these figures and tables (including the Supporting Online Material):

I have reviewed, or will review, the revised manuscript and approve of its submission to *Biomaterials Research* for publication.

☑ Yes ☐ No

If my university or institution has a separate publication license that applies to me (as required by Harvard, MIT, Open University, *et al.*) I have applied for a waiver.

☐ Yes ☑ There are no publication policies or restrictions limiting my ability to grant exclusive publication rights to The Korean Society for Biomaterials ("KSBM").

II. Data sharing:

I affirm that all data necessary for a reader of *Biomaterials Research* to understand and evaluate the conclusions of the paper will be archived in an approved database and made available to any reader.

☑ Yes ☐ No

III. Materials sharing:

I understand that after publication, all reasonable requests for materials and data must be fulfilled.

☑ Yes ☐ No

Biomaterials Research must be informed of any restrictions on sharing of materials [Materials Transfer Agreements (MTAs) or patents, for example] applying to materials used in the reported research.

☑ No, there are no MTAs

☐ Yes. Information on MTAs is described below.

IV. Conflict of Interest:

Biomaterials Research has a primary responsibility to its readers and to the public to provide in its pages clear and unbiased scientific results and analyses. We think that our readers should be informed of additional relationships of our authors that could pose a conflict of interest. Thus, for readers to evaluate the data and opinions presented in this journal, they must be informed of financial and other interests of our authors that may be at odds with unbiased presentation of data or analysis.

Therefore, *Biomaterials Research* believes that manuscripts should be accompanied by clear disclosures from all authors of their affiliations, funding sources, or financial holdings that might raise questions about possible sources of bias.
Disclosure is accomplished in three ways:

First, by a complete listing of the current institutional affiliations of the authors.

This list must include academic as well as corporate and other industrial affiliations. As the editors deem appropriate, items in this list will be included in the author affiliations printed in the manuscript. Please indicate below:
☑ Yes, all my affiliations are listed on the title page of the paper.

Additional affiliations not on the title page are:

Second, through the acknowledgment of all financial contributions to the work being reported, including contributions "in kind." All funding sources will be listed in the published manuscript. Please indicate below:

☑ All my funding sources for this study are listed in the acknowledgement section of the paper.
☐ Additional funding sources not noted in the manuscript are:

Third, through the execution of a statement disclosing to the Editors all financial holdings, professional affiliations, advisory positions, board memberships, patent holdings and the like that might bear a relationship to the subject matter of the contribution. The Editors will determine whether the material disclosed to them should be published as part of the article.

The following are declarable relationships: **Financial**: Significant financial interest (equity holdings or stock options) in any corporate entity dealing with the material or the subject matter of this contribution. Please disclose the entity and the nature and amount of the holding:

☑ None

☐ I have a financial relationship, as described below.

Management/Advisory affiliations: Within the last 3 years, status as an officer, a member of the Board, or a member of an Advisory Committee of any entity engaged in activity related to the subject matter of this contribution. Please disclose the nature of these relationships and the financial arrangements.

☑ None

☐ I have a management/advisory relationship, as described below:

Paid Consulting: Within the last 3 years, receipt of consulting fees, honoraria, speaking fees, or expert testimony fees from entities that have a financial interest in the results and materials of this study. Please enumerate.

☑ None

☐ I have a consulting relationship, as described below:

Patents: A planned, pending, or awarded patent on this work by any of the authors or their institutions. Please explain.

☑ None

☐ I or my institution has a patent related to this work, as described below

Declaration: I declare that I have read *Biomaterials Research's* full Conflict of Interest Policy and have disclosed all declarable relationships as defined therein, if any.

This form was submitted on 08/01/2024

Name Sehoon Moon

Signature

Biomaterials Research
A SCIENCE PARTNER JOURNAL

Authorship Form and Statement of Conflicts of Interest

Author Name: Hojeong lee

Manuscript Number: BMR-D-24-00200

Manuscript Title: Strategies to overcome hurdles in cancer immunotherapy

Each author must complete the following form prior to acceptance of their paper:

I. Authorship:

The authorship policies of *Biomaterials Research* follow those recommended by the report "On Being a Scientist", 3rd Edition, published by the US National Academy of Sciences (http://www.nap.edu/catalog/12192.html). In particular, we note that "just providing laboratory space for a project or furnishing a sample used in the research is not sufficient to be included as an author, although such contributions... may be recognized in a separate acknowledgment section."

In order to meet our requirements for authorship of a paper, you must have participated significantly in the reported research or writing of the paper. Please affirm that you meet these criteria by indicating your contribution to all of the following descriptions (from 0 to 100%):

**Authorship Activity**	**Level of participation**					
	0%	20%	40%	60%	80%	100%
Participated in the design and/or interpretation of the reported experiments or results.	○	○	●	○	○	○
Participated in the acquisition and/or analysis of data.<br>Indicate which data: ______	●	○	○	○	○	○
Participated in drafting and/or revising the manuscript.	○	○	●	○	○	○
Primarily responsible for a particular, specialized role in the research (e.g. statistical analysis, crystallography, preparation of cell lines, etc)<br>Briefly state roles: ______	●	○	○	○	○	○
Provided administrative, technical or supervisory support.	●	○	○	○	○	○

The senior author from each lab or group must answer this question: I have personally checked all the original data that was generated by my lab or group:

☑ Yes ☐ Not applicable; I am not the senior author or lab head.

If yes, these data are presented in these figures and tables (including the Supporting Online Material):

I have reviewed, or will review, the revised manuscript and approve of its submission to *Biomaterials Research* for publication.

☑ Yes ☐ No

If my university or institution has a separate publication license that applies to me (as required by Harvard, MIT, Open University, *et al.*) I have applied for a waiver.

☐ Yes ☑ There are no publication policies or restrictions limiting my ability to grant exclusive publication rights to The Korean Society for Biomaterials ("KSBM").

II. Data sharing:

I affirm that all data necessary for a reader of *Biomaterials Research* to understand and evaluate the conclusions of the paper will be archived in an approved database and made available to any reader.

☑ Yes ☐ No

III. Materials sharing:

I understand that after publication, all reasonable requests for materials and data must be fulfilled.

☑ Yes ☐ No

Biomaterials Research must be informed of any restrictions on sharing of materials [Materials Transfer Agreements (MTAs) or patents, for example] applying to materials used in the reported research.

☑ No, there are no MTAs

☐ Yes. Information on MTAs is described below.

IV. Conflict of Interest:

Biomaterials Research has a primary responsibility to its readers and to the public to provide in its pages clear and unbiased scientific results and analyses. We think that our readers should be informed of additional relationships of our authors that could pose a conflict of interest. Thus, for readers to evaluate the data and opinions presented in this journal, they must be informed of financial and other interests of our authors that may be at odds with unbiased presentation of data or analysis.

Therefore, *Biomaterials Research* believes that manuscripts should be accompanied by clear disclosures from all authors of their affiliations, funding sources, or financial holdings that might raise questions about possible sources of bias.
Disclosure is accomplished in three ways:

First, by a complete listing of the current institutional affiliations of the authors.

This list must include academic as well as corporate and other industrial affiliations. As the editors deem appropriate, items in this list will be included in the author affiliations printed in the manuscript. Please indicate below:
☑ _Yes, all my affiliations are listed on the title page of the paper.

Additional affiliations not on the title page are:

Second, through the acknowledgment of all financial contributions to the work being reported, including contributions "in kind." All funding sources will be listed in the published manuscript. Please indicate below:

☑ All my funding sources for this study are listed in the acknowledgement section of the paper.
☐ Additional funding sources not noted in the manuscript are:

Third, through the execution of a statement disclosing to the Editors all financial holdings, professional affiliations, advisory positions, board memberships, patent holdings and the like that might bear a relationship to the subject matter of the contribution. The Editors will determine whether the material disclosed to them should be published as part of the article.

The following are declarable relationships: **Financial**: Significant financial interest (equity holdings or stock options) in any corporate entity dealing with the material or the subject matter of this contribution. Please disclose the entity and the nature and amount of the holding:

☑ None

☐ I have a financial relationship, as described below.

Management/Advisory affiliations: Within the last 3 years, status as an officer, a member of the Board, or a member of an Advisory Committee of any entity engaged in activity related to the subject matter of this contribution. Please disclose the nature of these relationships and the financial arrangements.

☑ None

☐ I have a management/advisory relationship, as described below:

Paid Consulting: Within the last 3 years, receipt of consulting fees, honoraria, speaking fees, or expert testimony fees from entities that have a financial interest in the results and materials of this study. Please enumerate.

☑ None

☐ I have a consulting relationship, as described below:

Patents: A planned, pending, or awarded patent on this work by any of the authors or their institutions. Please explain.

☑ None

☐ I or my institution has a patent related to this work, as described below

Declaration: I declare that I have read *Biomaterials Research's* full Conflict of Interest Policy and have disclosed all declarable relationships as defined therein, if any.

This form was submitted on August 1st, 2024

Name Hojeong lee

Signature Hojeong lee 이호정

Biomaterials Research
A SCIENCE PARTNER JOURNAL

Authorship Form and Statement of Conflicts of Interest

Author Name: Juyong Lee

Manuscript Number: BMR-D-24-00200

Manuscript Title: Strategies to overcome hurdles in cancer immunotherapy

Each author must complete the following form prior to acceptance of their paper:

I. Authorship:

The authorship policies of *Biomaterials Research* follow those recommended by the report "On Being a Scientist", 3rd Edition, published by the US National Academy of Sciences (http://www.nap.edu/catalog/12192.html). In particular, we note that "just providing laboratory space for a project or furnishing a sample used in the research is not sufficient to be included as an author, although such contributions... may be recognized in a separate acknowledgment section."

In order to meet our requirements for authorship of a paper, you must have participated significantly in the reported research or writing of the paper. Please affirm that you meet these criteria by indicating your contribution to all of the following descriptions (from 0 to 100%):

Authorship Activity	Level of participation					
	0%	20%	40%	60%	80%	100%
Participated in the design and/or interpretation of the reported experiments or results.	○	○	●	○	○	○
Participated in the acquisition and/or analysis of data.<br>Indicate which data: ________	●	○	○	○	○	○
Participated in drafting and/or revising the manuscript.	○	○	●	○	○	○
Primarily responsible for a particular, specialized role in the research (e.g. statistical analysis, crystallography, preparation of cell lines, etc)<br>Briefly state roles: ________	●	○	○	○	○	○
Provided administrative, technical or supervisory support.	○	○	○	●	○	○

The senior author from each lab or group must answer this question: I have personally checked all the original data that was generated by my lab or group:

☑ Yes ☐ Not applicable; I am not the senior author or lab head.

If yes, these data are presented in these figures and tables (including the Supporting Online Material):

I have reviewed, or will review, the revised manuscript and approve of its submission to *Biomaterials Research* for publication.

☑ Yes ☐ No

If my university or institution has a separate publication license that applies to me (as required by Harvard, MIT, Open University, *et al.*) I have applied for a waiver.

☐ Yes ☑ There are no publication policies or restrictions limiting my ability to grant exclusive publication rights to The Korean Society for Biomaterials ("KSBM").

II. Data sharing:

I affirm that all data necessary for a reader of *Biomaterials Research* to understand and evaluate the conclusions of the paper will be archived in an approved database and made available to any reader.

☑ Yes ☐ No

III. Materials sharing:

I understand that after publication, all reasonable requests for materials and data must be fulfilled.

☑ Yes ☐ No

Biomaterials Research must be informed of any restrictions on sharing of materials [Materials Transfer Agreements (MTAs) or patents, for example] applying to materials used in the reported research.

☑ No, there are no MTAs

☐ Yes. Information on MTAs is described below.

IV. Conflict of Interest:

Biomaterials Research has a primary responsibility to its readers and to the public to provide in its pages clear and unbiased scientific results and analyses. We think that our readers should be informed of additional relationships of our authors that could pose a conflict of interest. Thus, for readers to evaluate the data and opinions presented in this journal, they must be informed of financial and other interests of our authors that may be at odds with unbiased presentation of data or analysis.

Therefore, *Biomaterials Research* believes that manuscripts should be accompanied by clear disclosures from all authors of their affiliations, funding sources, or financial holdings that might raise questions about possible sources of bias.
Disclosure is accomplished in three ways:

First, by a complete listing of the current institutional affiliations of the authors.

This list must include academic as well as corporate and other industrial affiliations. As the editors deem appropriate, items in this list will be included in the author affiliations printed in the manuscript. Please indicate below:
☑ Yes, all my affiliations are listed on the title page of the paper.

Additional affiliations not on the title page are:

Second, through the acknowledgment of all financial contributions to the work being reported, including contributions "in kind." All funding sources will be listed in the published manuscript. Please indicate below:

☑ All my funding sources for this study are listed in the acknowledgement section of the paper.
☐ Additional funding sources not noted in the manuscript are:

Third, through the execution of a statement disclosing to the Editors all financial holdings, professional affiliations, advisory positions, board memberships, patent holdings and the like that might bear a relationship to the subject matter of the contribution. The Editors will determine whether the material disclosed to them should be published as part of the article.

The following are declarable relationships: **Financial**: Significant financial interest (equity holdings or stock options) in any corporate entity dealing with the material or the subject matter of this contribution. Please disclose the entity and the nature and amount of the holding:

☑ None

☐ I have a financial relationship, as described below.

Management/Advisory affiliations: Within the last 3 years, status as an officer, a member of the Board, or a member of an Advisory Committee of any entity engaged in activity related to the subject matter of this contribution. Please disclose the nature of these relationships and the financial arrangements.

☑ None

☐ I have a management/advisory relationship, as described below:

Paid Consulting: Within the last 3 years, receipt of consulting fees, honoraria, speaking fees, or expert testimony fees from entities that have a financial interest in the results and materials of this study. Please enumerate.

☑ None

☐ I have a consulting relationship, as described below:

Patents: A planned, pending, or awarded patent on this work by any of the authors or their institutions. Please explain.

☑ None

☐ I or my institution has a patent related to this work, as described below

Declaration: I declare that I have read *Biomaterials Research's* full Conflict of Interest Policy and have disclosed all declarable relationships as defined therein, if any.

This form was submitted on August 1st, 2024

Name Juyong Lee

Signature

Biomaterials Research
A SCIENCE PARTNER JOURNAL

Authorship Form and Statement of Conflicts of Interest

Author Name: Byung-Soo Kim

Manuscript Number: BMR-D-24-00200

Manuscript Title: Strategies to overcome hurdles in cancer immunotherapy

Each author must complete the following form prior to acceptance of their paper:

I. Authorship:

The authorship policies of *Biomaterials Research* follow those recommended by the report "On Being a Scientist", 3rd Edition, published by the US National Academy of Sciences (http://www.nap.edu/catalog/12192.html). In particular, we note that "just providing laboratory space for a project or furnishing a sample used in the research is not sufficient to be included as an author, although such contributions... may be recognized in a separate acknowledgment section."

In order to meet our requirements for authorship of a paper, you must have participated significantly in the reported research or writing of the paper. Please affirm that you meet these criteria by indicating your contribution to all of the following descriptions (from 0 to 100%):

**Authorship Activity**	**Level of participation**					
	0%	20%	40%	60%	80%	100%
Participated in the design and/or interpretation of the reported experiments or results.	○	○	●	○	○	○
Participated in the acquisition and/or analysis of data.<br>Indicate which data: ______	●	○	○	○	○	○
Participated in drafting and/or revising the manuscript.	○	○	●	○	○	○
Primarily responsible for a particular, specialized role in the research (e.g. statistical analysis, crystallography, preparation of cell lines, etc)<br>Briefly state roles: ______	●	○	○	○	○	○
Provided administrative, technical or supervisory support.	○	○	○	●	○	○

The senior author from each lab or group must answer this question: I have personally checked all the original data that was generated by my lab or group:

☑ Yes ☐ Not applicable; I am not the senior author or lab head.

If yes, these data are presented in these figures and tables (including the Supporting Online Material):

I have reviewed, or will review, the revised manuscript and approve of its submission to *Biomaterials Research* for publication.

☑ Yes ☐ No

If my university or institution has a separate publication license that applies to me (as required by Harvard, MIT, Open University, *et al.*) I have applied for a waiver.

☐ Yes ☑ There are no publication policies or restrictions limiting my ability to grant exclusive publication rights to The Korean Society for Biomaterials ("KSBM").

II. Data sharing:

I affirm that all data necessary for a reader of *Biomaterials Research* to understand and evaluate the conclusions of the paper will be archived in an approved database and made available to any reader.

☑ Yes ☐ No

III. Materials sharing:

I understand that after publication, all reasonable requests for materials and data must be fulfilled.

☑ Yes ☐ No

Biomaterials Research must be informed of any restrictions on sharing of materials [Materials Transfer Agreements (MTAs) or patents, for example] applying to materials used in the reported research.

☑ No, there are no MTAs

☐ Yes. Information on MTAs is described below.

IV. Conflict of Interest:

Biomaterials Research has a primary responsibility to its readers and to the public to provide in its pages clear and unbiased scientific results and analyses. We think that our readers should be informed of additional relationships of our authors that could pose a conflict of interest. Thus, for readers to evaluate the data and opinions presented in this journal, they must be informed of financial and other interests of our authors that may be at odds with unbiased presentation of data or analysis.

Therefore, *Biomaterials Research* believes that manuscripts should be accompanied by clear disclosures from all authors of their affiliations, funding sources, or financial holdings that might raise questions about possible sources of bias.
Disclosure is accomplished in three ways:

First, by a complete listing of the current institutional affiliations of the authors.

This list must include academic as well as corporate and other industrial affiliations. As the editors deem appropriate, items in this list will be included in the author affiliations printed in the manuscript. Please indicate below:
☑ _Yes, all my affiliations are listed on the title page of the paper.

Additional affiliations not on the title page are:

Second, through the acknowledgment of all financial contributions to the work being reported, including contributions "in kind." All funding sources will be listed in the published manuscript. Please indicate below:

☑ All my funding sources for this study are listed in the acknowledgement section of the paper.
☐ Additional funding sources not noted in the manuscript are:

I have added my funding source (RS-2024-00354235) in revised manuscript

Third, through the execution of a statement disclosing to the Editors all financial holdings, professional affiliations, advisory positions, board memberships, patent holdings and the like that might bear a relationship to the subject matter of the contribution. The Editors will determine whether the material disclosed to them should be published as part of the article.

The following are declarable relationships: **Financial**: Significant financial interest (equity holdings or stock options) in any corporate entity dealing with the material or the subject matter of this contribution. Please disclose the entity and the nature and amount of the holding:

☑ None

☐ I have a financial relationship, as described below.

Management/Advisory affiliations: Within the last 3 years, status as an officer, a member of the Board, or a member of an Advisory Committee of any entity engaged in activity related to the subject matter of this contribution. Please disclose the nature of these relationships and the financial arrangements.

☑ None

☐ I have a management/advisory relationship, as described below:

Paid Consulting: Within the last 3 years, receipt of consulting fees, honoraria, speaking fees, or expert testimony fees from entities that have a financial interest in the results and materials of this study. Please enumerate.

☑ None

☐ I have a consulting relationship, as described below:

Patents: A planned, pending, or awarded patent on this work by any of the authors or their institutions. Please explain.

☑ None

☐ I or my institution has a patent related to this work, as described below

Declaration: I declare that I have read *Biomaterials Research's* full Conflict of Interest Policy and have disclosed all declarable relationships as defined therein, if any.

This form was submitted on 08/01/2024

Name Byung-Soo Kim

Signature [illegible]

Biomaterials Research
A SCIENCE PARTNER JOURNAL

Authorship Form and Statement of Conflicts of Interest

Author Name: Keehoon Jung

Manuscript Number: BMR-D-24-00200

Manuscript Title: Strategies to overcome hurdles in cancer immunotherapy

Each author must complete the following form prior to acceptance of their paper:

I. Authorship:

The authorship policies of *Biomaterials Research* follow those recommended by the report "On Being a Scientist", 3rd Edition, published by the US National Academy of Sciences (http://www.nap.edu/catalog/12192.html). In particular, we note that "just providing laboratory space for a project or furnishing a sample used in the research is not sufficient to be included as an author, although such contributions... may be recognized in a separate acknowledgment section."

In order to meet our requirements for authorship of a paper, you must have participated significantly in the reported research or writing of the paper. Please affirm that you meet these criteria by indicating your contribution to all of the following descriptions (from 0 to 100%):

Authorship Activity	Level of participation					
	0%	20%	40%	60%	80%	100%
Participated in the design and/or interpretation of the reported experiments or results.	○	○	●	○	●	○
Participated in the acquisition and/or analysis of data.<br><br>Indicate which data: ______	●	○	○	●	○	○
Participated in drafting and/or revising the manuscript.	○	○	●	○	●	○
Primarily responsible for a particular, specialized role in the research (e.g. statistical analysis, crystallography, preparation of cell lines, etc)<br><br>Briefly state roles: ______	●	○	○	●	○	○
Provided administrative, technical or supervisory support.	○	○	○	●	●	○

The senior author from each lab or group must answer this question: I have personally checked all the original data that was generated by my lab or group:

☑ Yes ☐ Not applicable; I am not the senior author or lab head.

If yes, these data are presented in these figures and tables (including the Supporting Online Material):

I have reviewed, or will review, the revised manuscript and approve of its submission to *Biomaterials Research* for publication.

☑ Yes ☐ No

If my university or institution has a separate publication license that applies to me (as required by Harvard, MIT, Open University, *et al.*) I have applied for a waiver.

☐ Yes ☑ There are no publication policies or restrictions limiting my ability to grant exclusive publication rights to The Korean Society for Biomaterials ("KSBM").

II. Data sharing:

I affirm that all data necessary for a reader of *Biomaterials Research* to understand and evaluate the conclusions of the paper will be archived in an approved database and made available to any reader.

☑ Yes ☐ No

III. Materials sharing:

I understand that after publication, all reasonable requests for materials and data must be fulfilled.

☑ Yes ☐ No

Biomaterials Research must be informed of any restrictions on sharing of materials [Materials Transfer Agreements (MTAs) or patents, for example] applying to materials used in the reported research.

☑ No, there are no MTAs

☐ Yes. Information on MTAs is described below.

IV. Conflict of Interest:

Biomaterials Research has a primary responsibility to its readers and to the public to provide in its pages clear and unbiased scientific results and analyses. We think that our readers should be informed of additional relationships of our authors that could pose a conflict of interest. Thus, for readers to evaluate the data and opinions presented in this journal, they must be informed of financial and other interests of our authors that may be at odds with unbiased presentation of data or analysis.

Therefore, *Biomaterials Research* believes that manuscripts should be accompanied by clear disclosures from all authors of their affiliations, funding sources, or financial holdings that might raise questions about possible sources of bias.
Disclosure is accomplished in three ways:

First, by a complete listing of the current institutional affiliations of the authors.

This list must include academic as well as corporate and other industrial affiliations. As the editors deem appropriate, items in this list will be included in the author affiliations printed in the manuscript. Please indicate below:
☑ _Yes, all my affiliations are listed on the title page of the paper.

Additional affiliations not on the title page are:

Second, through the acknowledgment of all financial contributions to the work being reported, including contributions "in kind." All funding sources will be listed in the published manuscript. Please indicate below:

☑ All my funding sources for this study are listed in the acknowledgement section of the paper.
☐ Additional funding sources not noted in the manuscript are:

Third, through the execution of a statement disclosing to the Editors all financial holdings, professional affiliations, advisory positions, board memberships, patent holdings and the like that might bear a relationship to the subject matter of the contribution. The Editors will determine whether the material disclosed to them should be published as part of the article.

The following are declarable relationships: **Financial**: Significant financial interest (equity holdings or stock options) in any corporate entity dealing with the material or the subject matter of this contribution. Please disclose the entity and the nature and amount of the holding:

☑ None

☐ I have a financial relationship, as described below.

Management/Advisory affiliations: Within the last 3 years, status as an officer, a member of the Board, or a member of an Advisory Committee of any entity engaged in activity related to the subject matter of this contribution. Please disclose the nature of these relationships and the financial arrangements.

☑ None

☐ I have a management/advisory relationship, as described below:

Paid Consulting: Within the last 3 years, receipt of consulting fees, honoraria, speaking fees, or expert testimony fees from entities that have a financial interest in the results and materials of this study. Please enumerate.

☑ None

☐ I have a consulting relationship, as described below:

Patents: A planned, pending, or awarded patent on this work by any of the authors or their institutions. Please explain.

☑ None

☐ I or my institution has a patent related to this work, as described below

Declaration: I declare that I have read *Biomaterials Research's* full Conflict of Interest Policy and have disclosed all declarable relationships as defined therein, if any.

This form was submitted on 08/01/2024

Name Keehoon Jung

Signature

Biomaterials Research
A SCIENCE PARTNER JOURNAL

Authorship Form and Statement of Conflicts of Interest

Author Name: Hyungseok Seo

Manuscript Number: BMR-D-24-00200

Manuscript Title: Strategies to overcome hurdles in cancer immunotherapy

Each author must complete the following form prior to acceptance of their paper:

I. Authorship:

The authorship policies of *Biomaterials Research* follow those recommended by the report "On Being a Scientist", 3rd Edition, published by the US National Academy of Sciences (http://www.nap.edu/catalog/12192.html). In particular, we note that "just providing laboratory space for a project or furnishing a sample used in the research is not sufficient to be included as an author, although such contributions... may be recognized in a separate acknowledgment section."

In order to meet our requirements for authorship of a paper, you must have participated significantly in the reported research or writing of the paper. Please affirm that you meet these criteria by indicating your contribution to all of the following descriptions (from 0 to 100%):

Authorship Activity	Level of participation					
	0%	20%	40%	60%	80%	100%
Participated in the design and/or interpretation of the reported experiments or results.	○	○	○	○	●	○
Participated in the acquisition and/or analysis of data.<br>Indicate which data: ________	○	○	○	○	●	○
Participated in drafting and/or revising the manuscript.	○	○	○	○	●	○
Primarily responsible for a particular, specialized role in the research (e.g. statistical analysis, crystallography, preparation of cell lines, etc)<br>Briefly state roles: ________	○	○	○	○	●	○
Provided administrative, technical or supervisory support.	○	○	○	○	○	●

The senior author from each lab or group must answer this question: I have personally checked all the original data that was generated by my lab or group:

☑ Yes ☐ Not applicable; I am not the senior author or lab head.

If yes, these data are presented in these figures and tables (including the Supporting Online Material):

Figure 1, 2 and Table 1, 2

I have reviewed, or will review, the revised manuscript and approve of its submission to *Biomaterials Research* for publication.

☑ Yes ☐ No

If my university or institution has a separate publication license that applies to me (as required by Harvard, MIT, Open University, *et al.*) I have applied for a waiver.

☐ Yes ☑ There are no publication policies or restrictions limiting my ability to grant exclusive publication rights to The Korean Society for Biomaterials ("KSBM").

II. Data sharing:

I affirm that all data necessary for a reader of *Biomaterials Research* to understand and evaluate the conclusions of the paper will be archived in an approved database and made available to any reader.

☑ Yes ☐ No

III. Materials sharing:

I understand that after publication, all reasonable requests for materials and data must be fulfilled.

☑ Yes ☐ No

Biomaterials Research must be informed of any restrictions on sharing of materials [Materials Transfer Agreements (MTAs) or patents, for example] applying to materials used in the reported research.

☑ No, there are no MTAs

☐ Yes. Information on MTAs is described below.

IV. Conflict of Interest:

Biomaterials Research has a primary responsibility to its readers and to the public to provide in its pages clear and unbiased scientific results and analyses. We think that our readers should be informed of additional relationships of our authors that could pose a conflict of interest. Thus, for readers to evaluate the data and opinions presented in this journal, they must be informed of financial and other interests of our authors that may be at odds with unbiased presentation of data or analysis.

Therefore, *Biomaterials Research* believes that manuscripts should be accompanied by clear disclosures from all authors of their affiliations, funding sources, or financial holdings that might raise questions about possible sources of bias.
Disclosure is accomplished in three ways:

First, by a complete listing of the current institutional affiliations of the authors.

This list must include academic as well as corporate and other industrial affiliations. As the editors deem appropriate, items in this list will be included in the author affiliations printed in the manuscript. Please indicate below:
☑ Yes, all my affiliations are listed on the title page of the paper.

Additional affiliations not on the title page are:

Second, through the acknowledgment of all financial contributions to the work being reported, including contributions "in kind." All funding sources will be listed in the published manuscript. Please indicate below:

☑ All my funding sources for this study are listed in the acknowledgement section of the paper.
☐ Additional funding sources not noted in the manuscript are:

I have added my funding sources(RS-2023-00242443, RS-2023-00282907) in revised manuscript

Third, through the execution of a statement disclosing to the Editors all financial holdings, professional affiliations, advisory positions, board memberships, patent holdings and the like that might bear a relationship to the subject matter of the contribution. The Editors will determine whether the material disclosed to them should be published as part of the article.

The following are declarable relationships: **Financial**: Significant financial interest (equity holdings or stock options) in any corporate entity dealing with the material or the subject matter of this contribution. Please disclose the entity and the nature and amount of the holding:

☑ None

☐ I have a financial relationship, as described below.

Management/Advisory affiliations: Within the last 3 years, status as an officer, a member of the Board, or a member of an Advisory Committee of any entity engaged in activity related to the subject matter of this contribution. Please disclose the nature of these relationships and the financial arrangements.

☑ None

☐ I have a management/advisory relationship, as described below:

Paid Consulting: Within the last 3 years, receipt of consulting fees, honoraria, speaking fees, or expert testimony fees from entities that have a financial interest in the results and materials of this study. Please enumerate.

☑ None

☐ I have a consulting relationship, as described below:

Patents: A planned, pending, or awarded patent on this work by any of the authors or their institutions. Please explain.

☑ None

☐ I or my institution has a patent related to this work, as described below

Declaration: I declare that I have read *Biomaterials Research's* full Conflict of Interest Policy and have disclosed all declarable relationships as defined therein, if any.

This form was submitted on 8/1/2024

Name Hyungseok Seo

Signature H. Seo 2024.07.31 21:56:57 +09'00'

Biomaterials Research
A SCIENCE PARTNER JOURNAL

Authorship Form and Statement of Conflicts of Interest

Author Name: Yeonseok Chung

Manuscript Number: BMR-D-24-00200

Manuscript Title: Strategies to overcome hurdles in cancer immunotherapy

Each author must complete the following form prior to acceptance of their paper:

I. Authorship:

The authorship policies of *Biomaterials Research* follow those recommended by the report "On Being a Scientist", 3rd Edition, published by the US National Academy of Sciences (http://www.nap.edu/catalog/12192.html). In particular, we note that "just providing laboratory space for a project or furnishing a sample used in the research is not sufficient to be included as an author, although such contributions... may be recognized in a separate acknowledgment section."

In order to meet our requirements for authorship of a paper, you must have participated significantly in the reported research or writing of the paper. Please affirm that you meet these criteria by indicating your contribution to all of the following descriptions (from 0 to 100%):

Authorship Activity	Level of participation					
	0%	20%	40%	60%	80%	100%
Participated in the design and/or interpretation of the reported experiments or results.	○	○	●	○	○	○
Participated in the acquisition and/or analysis of data.<br><br>Indicate which data: ______	●	○	○	○	○	○
Participated in drafting and/or revising the manuscript.	○	○	●	○	○	○
Primarily responsible for a particular, specialized role in the research (e.g. statistical analysis, crystallography, preparation of cell lines, etc)<br><br>Briefly state roles: ______	●	○	○	○	○	○
Provided administrative, technical or supervisory support.	○	○	○	●	○	○

The senior author from each lab or group must answer this question: I have personally checked all the original data that was generated by my lab or group:

☑ Yes ☐ Not applicable; I am not the senior author or lab head.

If yes, these data are presented in these figures and tables (including the Supporting Online Material):

I have reviewed, or will review, the revised manuscript and approve of its submission to *Biomaterials Research* for publication.

☑ Yes ☐ No

If my university or institution has a separate publication license that applies to me (as required by Harvard, MIT, Open University, *et al.*) I have applied for a waiver.

☐ Yes ☑ There are no publication policies or restrictions limiting my ability to grant exclusive publication rights to The Korean Society for Biomaterials ("KSBM").

II. Data sharing:

I affirm that all data necessary for a reader of *Biomaterials Research* to understand and evaluate the conclusions of the paper will be archived in an approved database and made available to any reader.

☑ Yes ☐ No

III. Materials sharing:

I understand that after publication, all reasonable requests for materials and data must be fulfilled.

☑ Yes ☐ No

Biomaterials Research must be informed of any restrictions on sharing of materials [Materials Transfer Agreements (MTAs) or patents, for example] applying to materials used in the reported research.

☑ No, there are no MTAs

☐ Yes. Information on MTAs is described below.

IV. Conflict of Interest:

Biomaterials Research has a primary responsibility to its readers and to the public to provide in its pages clear and unbiased scientific results and analyses. We think that our readers should be informed of additional relationships of our authors that could pose a conflict of interest. Thus, for readers to evaluate the data and opinions presented in this journal, they must be informed of financial and other interests of our authors that may be at odds with unbiased presentation of data or analysis.

Therefore, *Biomaterials Research* believes that manuscripts should be accompanied by clear disclosures from all authors of their affiliations, funding sources, or financial holdings that might raise questions about possible sources of bias.
Disclosure is accomplished in three ways:

First, by a complete listing of the current institutional affiliations of the authors.

This list must include academic as well as corporate and other industrial affiliations. As the editors deem appropriate, items in this list will be included in the author affiliations printed in the manuscript. Please indicate below:
☑ Yes, all my affiliations are listed on the title page of the paper.

Additional affiliations not on the title page are:

Second, through the acknowledgment of all financial contributions to the work being reported, including contributions "in kind." All funding sources will be listed in the published manuscript. Please indicate below:

☑ All my funding sources for this study are listed in the acknowledgement section of the paper.
☐ Additional funding sources not noted in the manuscript are:

Third, through the execution of a statement disclosing to the Editors all financial holdings, professional affiliations, advisory positions, board memberships, patent holdings and the like that might bear a relationship to the subject matter of the contribution. The Editors will determine whether the material disclosed to them should be published as part of the article.

The following are declarable relationships: **Financial**: Significant financial interest (equity holdings or stock options) in any corporate entity dealing with the material or the subject matter of this contribution. Please disclose the entity and the nature and amount of the holding:

- [x] None
- [ ] I have a financial relationship, as described below.

Management/Advisory affiliations: Within the last 3 years, status as an officer, a member of the Board, or a member of an Advisory Committee of any entity engaged in activity related to the subject matter of this contribution. Please disclose the nature of these relationships and the financial arrangements.

- [x] None
- [ ] I have a management/advisory relationship, as described below:

Paid Consulting: Within the last 3 years, receipt of consulting fees, honoraria, speaking fees, or expert testimony fees from entities that have a financial interest in the results and materials of this study. Please enumerate.

- [x] None
- [ ] I have a consulting relationship, as described below:

Patents: A planned, pending, or awarded patent on this work by any of the authors or their institutions. Please explain.

- [x] None
- [ ] I or my institution has a patent related to this work, as described below

Declaration: I declare that I have read *Biomaterials Research's* full Conflict of Interest Policy and have disclosed all declarable relationships as defined therein, if any.

This form was submitted on 08/01/2024

Name Yeonseok Chung

Signature Yeonseok Chung 디지털 서명자Yeonseok Chung
날짜: 2024.08.01 09:32:49
+09'00'
